# Insulin Signaling Regulates Fatty Acid Catabolism at the Level of CoA Activation

**DOI:** 10.1371/journal.pgen.1002478

**Published:** 2012-01-19

**Authors:** Xiaojun Xu, Peddinti Gopalacharyulu, Tuulikki Seppänen-Laakso, Anna-Liisa Ruskeepää, Cho Cho Aye, Brian P. Carson, Silvia Mora, Matej Orešič, Aurelio A. Teleman

**Affiliations:** 1 German Cancer Research Center (DKFZ), Heidelberg, Germany; 2 VTT Technical Research Centre of Finland, Espoo, Finland; 3 Department of Cellular and Molecular Physiology, Institute of Translational Medicine, University of Liverpool, Liverpool, United Kingdom; University of California San Francisco, United States of America

## Abstract

The insulin/IGF signaling pathway is a highly conserved regulator of metabolism in flies and mammals, regulating multiple physiological functions including lipid metabolism. Although insulin signaling is known to regulate the activity of a number of enzymes in metabolic pathways, a comprehensive understanding of how the insulin signaling pathway regulates metabolic pathways is still lacking. Accepted knowledge suggests the key regulated step in triglyceride (TAG) catabolism is the release of fatty acids from TAG via the action of lipases. We show here that an additional, important regulated step is the activation of fatty acids for beta-oxidation via Acyl Co-A synthetases (ACS). We identify *pudgy* as an ACS that is transcriptionally regulated by direct FOXO action in *Drosophila*. Increasing or reducing *pudgy* expression *in vivo* causes a decrease or increase in organismal TAG levels respectively, indicating that *pudgy* expression levels are important for proper lipid homeostasis. We show that multiple ACSs are also transcriptionally regulated by insulin signaling in mammalian cells. In sum, we identify fatty acid activation onto CoA as an important, regulated step in triglyceride catabolism, and we identify a mechanistic link through which insulin regulates lipid homeostasis.

## Author Summary

Type 2 diabetes, which is reaching epidemic proportions worldwide, is often associated with obesity and an imbalance in organismal lipid homeostasis. Therefore, understanding how insulin regulates lipid biosynthesis and breakdown is necessary. Surprisingly, the molecular mechanisms by which insulin regulates fatty acid catabolism are not entirely understood. We show here that insulin signaling regulates expression of acyl-CoA Synthetases (ACS). ACSs couple fatty acids to Coenzyme A, thereby activating them for subsequent biochemical reactions. In Drosophila, we find that insulin signaling modulates expression of one ACS called Pudgy, which activates fatty acids for beta-oxidation. Modulation of pudgy expression leads to changes in overall organismal lipid homeostasis. Likewise, we show that in mammalian cells insulin signaling regulates expression of a number of ACSs and that ACS expression modulates steady-state lipid levels.

## Introduction

The insulin/IGF signaling (IIS) pathway is a highly conserved and critical regulator of metabolism in mammals and in flies, where it senses organismal nutrient levels to regulate multiple physiological functions including carbohydrate metabolism, tissue growth and longevity [1]–[3]. Insulin regulates carbohydrate metabolism by controlling expression and activity of a number of metabolic enzymes such as phosphofructokinase-2, PEPCK, Glycogen synthase and Glycogen phosphorylase [4]. Conditions of altered insulin signaling are associated not only with changes in carbohydrate metabolism, but also with abnormal lipid metabolism, as in the cases of Type 2 Diabetes-associated obesity and Non-Alcoholic Hepatic Steatosis [5], [6]. A large body of evidence suggests that insulin resistance plays a central, causal role in the development of the lipid imbalances observed in both of these conditions [5], [6], however the molecular mechanisms leading to these lipid imbalances are not completely understood. This raises the need to better understand the molecular connections between insulin signaling and lipid metabolism.

**Figure 1:**
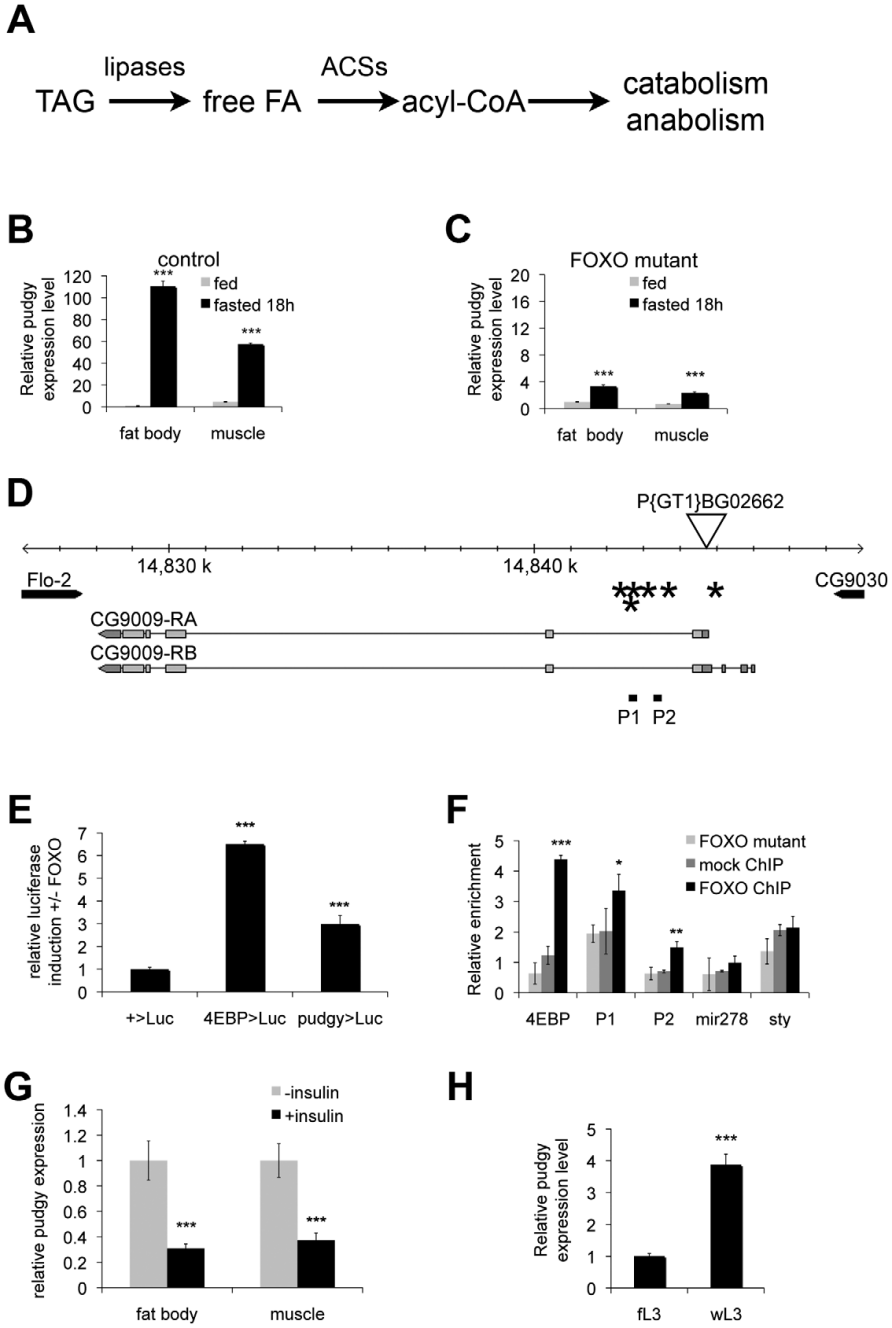
*Pudgy* is a direct FOXO target, upregulated upon fasting. (A) Simplified schematic of triacylglycerol catabolism. Triacylglycerides (TAG) are cleaved by lipases, releasing free fatty acids (free FA). These are linked to CoA by acyl-CoA synthetases (ACSs). Acyl-CoA moities are then either oxidized via beta-oxidation or used for biosynthetic reactions. (B,C) *pudgy* expression is upregulated in a FOXO-dependent manner upon nutrient withdrawal in 3^rd^ instar larvae. Control (B) or FOXO^21/25^ null mutants (C) were either fed or deprived of food for 18 hours, and expression of *pudgy* in fat body or muscle analyzed by quantitative RT-PCR relative to *rp49*. (D) Schematic of *pudgy* (*pdgy*, CG9009) genomic locus. Site of the P-element P{GT1}BG02662 insertion in the 5′UTR of *pdgy* is indicated. Asterisks: FOXO binding sites. P1 and P2: amplicons tested in the FOXO chromatin IP in panel F. (E) Luciferase assay testing FOXO-responsiveness of genomic enhancers. Genomic fragments containing the FOXO enhancer of the bona-fide FOXO target 4E-BP (4EBP>Luc), or containing three of the FOXO binding sites in *pudgy* intron 1 (pudgy>Luc) were introduced into a luciferase vector containing a basal promoter and firefly luciferase (+>Luc). Relative luciferase induction in the presence versus absence of FOXO expression is indicated, normalized to a renilla luciferase control. (F) FOXO binds the *pudgy* promoter region. Quantification by Q-PCR of chromatin immunoprecipitated (ChIP) material from *FOXO* mutant animals using anti-FOXO antibody (“FOXO mutant”, a negative control) or from wildtype animals using either pre-immune serum (“mock ChIP”, a negative control) or anti-FOXO antibody (“FOXO ChIP”). Promoter regions assayed were those of *4E-BP* (a direct FOXO target), *mir-278* and *sty* (two negative controls) and two regions of the first intron of *pudgy*, P1 and P2, as indicated in Panel D. (G) Insulin signaling represses *pudgy* expression in fat body and muscle. *Pudgy* expression in tissues explanted from feeding 3rd instar larvae treated in Schneider's medium with or without 10 µg/ml insulin for 30 min, quantified by Q-RT-PCR relative to *rp49*. (H) *pudgy* expression increases in third instar larvae upon wandering. *pudgy* mRNA levels measured by quantitative RT-PCR relative to *rp49* for feeding (“fL3”) and wandering (“wL3”) 3^rd^ instar larvae. For all panels, Error bars: Std. Dev., * ttest<0.05, ** ttest<0.01, ***ttest<0.001.

The molecular relationship between insulin signaling and lipid homeostasis is complex, as dyslipidemia is considered to be both a cause and a consequence of insulin resistance [6]. That said, IIS clearly plays a causative role in regulating the balance of lipid production versus breakdown in animals, since mice and flies in which IIS has been specifically manipulated have altered lipid metabolism ([7], [8] and reviewed in [9]–[11]). The molecular mechanisms by which IIS regulates lipid metabolism are only partially understood. On the one hand, IIS promotes fatty acid biosynthesis [12], [13]. On the other, IIS regulates fatty acid catabolism [13], [14]. Fatty acid catabolism is a multi-step process (Figure 1A). First, fatty acids are mobilized from stored triacylglycerols (TAG) via the activity of lipases to yield free fatty acids. Second, the free fatty acids are activated by coupling to Coenzyme A (CoA). This step is catalyzed by the acyl-CoA synthetase (ACS) family of enzymes. Third, the free fatty acids are imported into mitochondria. Finally, in mitochondria, the fatty acids are oxidized, yielding energy. Some of the steps in this catabolic pathway are known to be regulated by IIS. For instance, IIS inhibits expression and activity of lipases such as adipose triglyceride lipase and hormones sensitive lipase [15], [16]. IIS also decreases the rate of fatty acid entry into mitochondria [17] in part via a FoxO-dependent process [18]. A complete molecular understanding of how IIS regulates fatty acid catabolism, however, is currently lacking.

The upstream signaling events of the IIS pathway are fairly well characterized. Activation of insulin/IGF receptor(s) leads to a relay of phosphorylation events activating a number of kinases including PI3K, Akt/PKB, TOR-C1 and S6K, thereby inhibiting a key transcription factor FOXO (for review [19]). A challenge in the field remains, however, to obtain a complete understanding of how these upstream ‘signaling’ components of the IIS pathway link to, and regulate, the metabolic biochemical pathways controlling cellular metabolism. Discovering the connections between the signaling components of the insulin pathway and the metabolic enzymes controlling cellular biochemical pathways remains an important step in understanding how IIS controls metabolism generally, and lipid metabolism in particular.

We identify here an ACS which we term pudgy, as a gene that is strongly upregulated upon fasting in Drosophila. We find that *pudgy* is a target of the insulin signaling pathway, as its expression is suppressed by insulin signaling, as a consequence of direct regulation by FOXO. We find that animals with reduced levels of *pudgy* expression are hyper-triglyceridemic and have defects in their lipid usage upon fasting. This suggests that in order to effectively channel fatty acids towards beta-oxidation upon fasting conditions, organisms need to induce both the lipolysis of fatty acids from TAG, as well as the activation of fatty acids at mitochondria for beta-oxidation. Finally, we show that expression of multiple mammalian ACSs are also regulated by insulin signaling in mouse muscle, liver and adipose cells. In sum, this work uncovers fatty acid activation by ACSs as a novel and important insulin-regulated step in TAG catabolism.

## Results

### The Acyl-CoA Synthetase pudgy is a direct FOXO target

We previously studied the transcriptional output of insulin signaling in Drosophila by performing microarray analyses on fasted versus fed animals [20]. By comparing wildtype versus FOXO mutant animals, we pinpointed genes that are regulated in a FOXO-dependent manner [20]. In this and similar studies by other groups [21]–[24], a number of acyl-CoA synthetases (ACSs) were found to be regulated by nutrient status. In particular, the ACS gene CG9009 emerged in our analysis as a strongly regulated gene, which we characterize further here.

Quantitative RT-PCR analysis on wildtype larvae shows that expression of CG9009, which we term here *pudgy* (*pdgy*), is very strongly up-regulated in the fat body upon 18 hours of fasting, increasing 110-fold (Figure 1B). (The Drosophila fat body performs the functions of mammalian adipose tissue and liver combined.) In contrast, in FOXO^21/25^ null mutant larvae, expression of *pudgy* only increases 3.4-fold in the fat body upon fasting, indicating that the up-regulation of *pudgy* is strongly FOXO dependent (Figure 1C, note different scale compared to Figure 1B). *Pudgy* expression behaved similarly in muscle (Figures 1B and 1C).

*Pudgy* expression could either be regulated directly or indirectly by FOXO. To distinguish these possibilities, we performed a bioinformatic scan of the promoter region of the *pudgy* gene, as we previously showed that functional FOXO binding sites in Drosophila are usually clustered within a few kilobases of the transcription start site of regulated genes [20]. The *pudgy* promoter region had a significant number of consensus FOXO binding sites – 3 perfect (GTAAACAA) and 3 imperfect (1 mismatch in the 1^st^ or 2^nd^ position) (indicated by asterisks in Figure 1D). We first tested whether this region is able to serve as a FOXO-responsive cis-regulatory enhancer element. Test genomic regions were linked to a basal promoter directing luciferase expression in S2 cells. As a positive control, a genomic region of the *4E-BP* gene, an established direct target of FOXO [25], [26], was able to induce luciferase activity in response to FOXO expression (Figure 1E). Likewise, an 800 bp fragment of the *pudgy* region, containing 3 of the 6 FOXO binding sites, induced luciferase activity in response to FOXO expression (Figure 1E), suggesting it is a bona fide FOXO response element. Next, to test whether endogenous FOXO binds these sites *in vivo*, we performed chromatin immunoprecipitations (ChIP) of endogenous FOXO from 3^rd^ instar larvae. We performed two negative controls: a mock ChIP using pre-immune serum on wildtype larval lysates, as well as a ChIP using anti-FOXO antibody [26] on lysates of *FOXO^21/25^* null mutant larvae (Figure 1F) [23]. Quantitative PCR on the immunoprecipitated material revealed that the promoter region of *4E-BP* was strongly enriched in the FOXO ChIP from wildtype larvae compared to the negative control ChIPs (ttest<0.001, Figure 1F, black bars versus grey bars). Likewise, two test regions in the first intron of *pudgy*, P1 and P2 (Figure 1D), were also significantly enriched in the FOXO ChIP compared to the negative control ChIPs (ttest<0.05 for P1 and ttest<0.01 for P2, Figure 1F). As negative controls, the genomic regions of *mir-278* and *sty* were not enriched in the FOXO ChIP compared to control ChIPs (Figure 1F). Together, these data indicate that FOXO binds the *pudgy* promoter region *in vivo*. In sum, this identifies *pudgy* is a bona fide direct FOXO target.

Since FOXO activity is repressed by insulin signaling, *pudgy* expression should also be repressed by insulin. Indeed, *pudgy* expression was reduced in explants of both fat body tissue and muscle tissue when they were treated with insulin (ttest<0.001, Figure 1G). Moreover, *in vivo*, insulin signaling drops when larvae have terminated feeding and start wandering out of the food. Consistent with this, *pudgy* expression was 4-fold higher in wandering 3^rd^ instar larvae (wL3) compared to feeding 3^rd^ instar larvae (fL3) (Figure 1H).

### Pudgy is an Acyl-CoA Synthetase associated with mitochondria

Previous computational analyses identified CG9009/*pudgy* as a gene encoding an acyl-CoA synthetase (ACS) [27]. ACSs are a family of enzymes which activate free fatty acids for subsequent anabolic or catabolic reactions by loading them onto CoA. Each member of this family has distinct substrate specificity, loading fatty acid molecules of different lengths or saturation onto CoA [27]. In addition, each member of the ACS family has a distinct intracellular localization. This is particularly relevant in lieu of that fact that subsequent reactions involving activated acyl-CoA molecules take place in distinct subcellular compartments. Fatty acid oxidation occurs either in mitochondria in the form of beta-oxidation, or in peroxisomes. In contrast, anabolic reactions take place predominantly in the cytoplasm or endoplasmic reticulum. Thus the subcellular localization of each member of the ACS family may influence the fate of the acyl-CoA molecules that it generates [28]. By channeling fatty acids towards downstream anabolic or catabolic processes, ACSs such as Pudgy have the potential to influence the fate of the fatty acids and the overall balance of organismal lipid homeostasis [29], a hypothesis which we test here.

**Figure 2:**
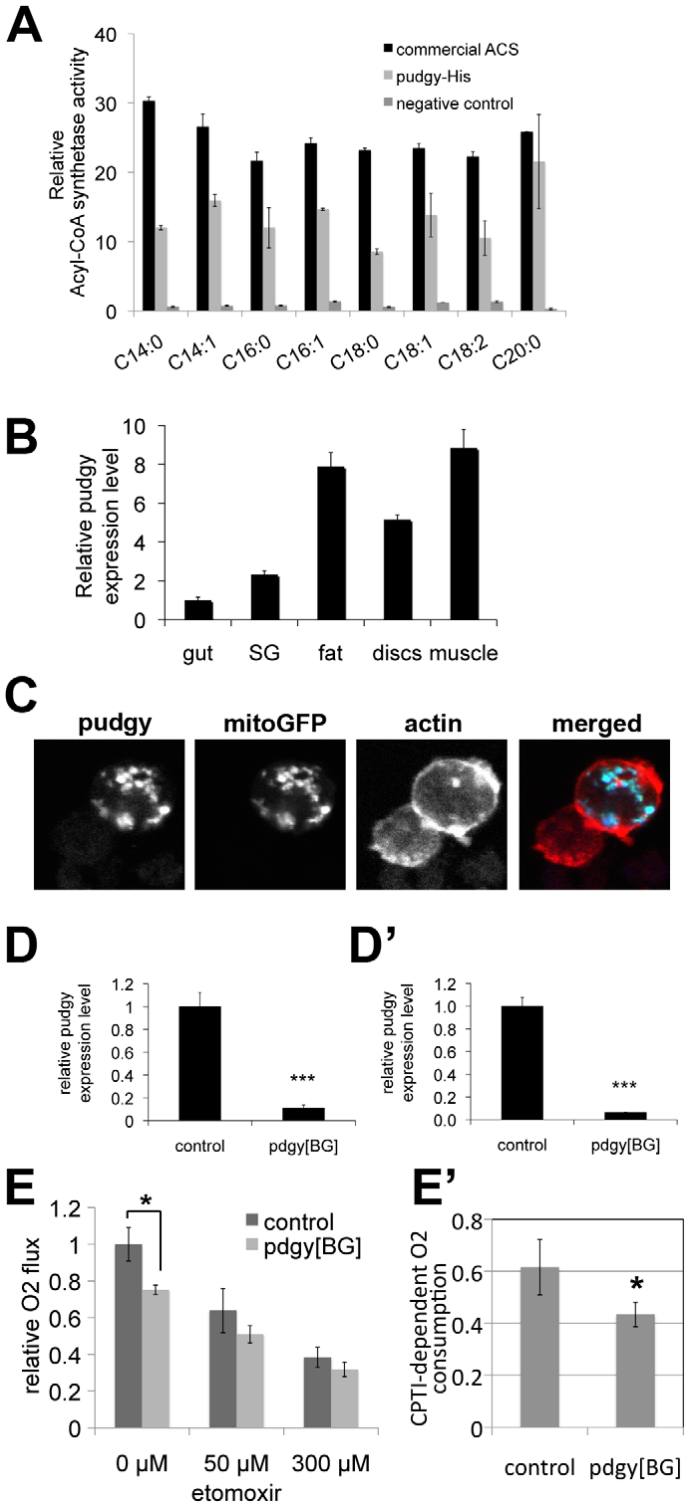
*Pudgy* is an ACS localized to mitochondria influencing lipid oxidation. (A) Recombinant *pudgy* protein exhibits ACS activity in vitro. Equal amounts of recombinant, commercial ACS from the Free Fatty Acids Quantification Kit (Biovision) (“commercial ACS”), purified recombinant His-tagged pudgy protein (“pudgy-His”), or an equivalent amount of eluate from a parallel purification with bacteria not expressing pudgy-His (“negative control”), were mixed with free fatty acids and CoA in vitro. The rate of synthesis of acyl-CoA is indicated. All ACS activities for commercial ACS and pudgy-His were significantly above negative control background (ttest<0.05). (B) *pudgy* expression measured by Q-RT-PCR relative to *rp49* in various tissues of wildtype 3rd instar larvae, as indicated. SG: salivary gland. (C) Pudgy localizes to mitochondria. Immunofluorescence micrograph of S2 cells transfected to express *pudgy-HA* (blue) and mito-GFP to mark mitochondria (green) shows very good co-localization of the two proteins. Actin staining (red) delineates the cell outline. (Pearson's correlation = 0.84±0.04 on 8 images [50]). (D–D′) *pudgy* expression, by Q-RT-PCR relative to *rp49*, in control w^1118^ or *pdgy[BG]* mutant feeding 3^rd^ instar larvae (96 h AEL) (D) or adult males (D′). (E) Lipid oxidation in *pdgy[BG]* mutants is impaired. Oxygen consumption rate, measured using a Clark electrode, is significantly reduced in *pdgy[BG]* mutant larvae (light bars) compared to controls (dark bars) (*ttest = 0.03). This difference is abrogated in the presence of the CPT1 inhibitor etomoxir, indicating it is due to a difference in lipid oxidation. (E′) *pdgy[BG]* mutants have significantly reduced CPTI-dependent oxygen consumption. CPTI-dependent O_2_ consumption was calculated by subtracting the rate of oxygen consumption in the presence of 300 µM etomoxir (ie CPTI independent) from the total rate of oxygen consumption in the absence of drug.

To confirm that the protein encoded by *pudgy* is indeed an ACS, we recombinantly expressed and purified His-tagged pudgy from E. coli and found that it has acyl-CoA synthetase activity in vitro (Figure 2A). *Pudgy* is expressed in all tissues of the larva that we tested (Figure 2B). Since the localization of ACSs influences their function, we investigated the subcellular localization of pudgy. Expression of a C-terminal epitope-tagged version of pudgy in S2 cells revealed that it co-localizes with a GFP construct marking mitochondria (mitoGFP) (Figure 2C), suggesting pudgy may load fatty acids onto CoA for mitochondrial beta-oxidation (see below). To study the physiological role of pudgy, we obtained flies containing a transposon insertion in the 5′ UTR of *pudgy* (P{GT1}BG02662, “*pdgy[BG]*”, Figure 1D). The *pdgy[BG]* mutation was back-crossed into the w^1118^ background for five generations (via females) in order to obtain two stocks with similar genetic backgrounds, differing by presence or absence of the *pdgy[BG]* mutation. The resulting stock carrying the *pdgy[BG]* mutation in the w^1118^ background was used for all subsequent experiments described here, and will be referred to as *pdgy[BG]* mutant flies, whereas the w^1118^ flies will be referred to as controls. *pdgy[BG]* homozygous larvae and adults have strongly reduced expression of *pudgy*, measured by quantitative RT-PCR (Figure 2D and 2D′ respectively). We believe this animal model may not represent a complete *pudgy* null situation, but is a good model for studying the physiological effects of strongly reduced *pudgy* function. To test whether pudgy is involved in fatty acid oxidation, we measured oxygen consumption in control and *pdgy[BG]* mutant larvae using a Clark electrode. In the absence of drugs, oxygen consumption in *pdgy[BG]* mutant larvae was significantly reduced compared to controls (Figure 2E). Subsequent addition of etomoxir, a specific inhibitor of Carnitine palmitoyltransferase I (CPTI) [30], required for the transport of fatty acids into mitochondria where beta-oxidation takes place, causes this difference in oxygen consumption to be abrogated (300 µM etomoxir, Figure 2E). This indicates that the difference in oxygen consumption between *pdgy[BG]* mutants and controls is due to differential mitochondrial lipid oxidation. Subtraction of the basal rate of oxygen consumption in the presence of 300 µM etomoxir from the oxygen consumption in the absence of etomoxir, yields the rate of CPTI-dependent oxygen consumption, revealing that *pdgy[BG]* mutants have significantly reduced ß-oxidation levels compared to controls (Figure 2E′). Conversely, overexpression of pudgy in larvae was sufficient to increase the rate of fatty acid beta-oxidation (Figure S1).

### Pudgy expression levels regulate organismal lipid homeostasis

**Figure 3:**
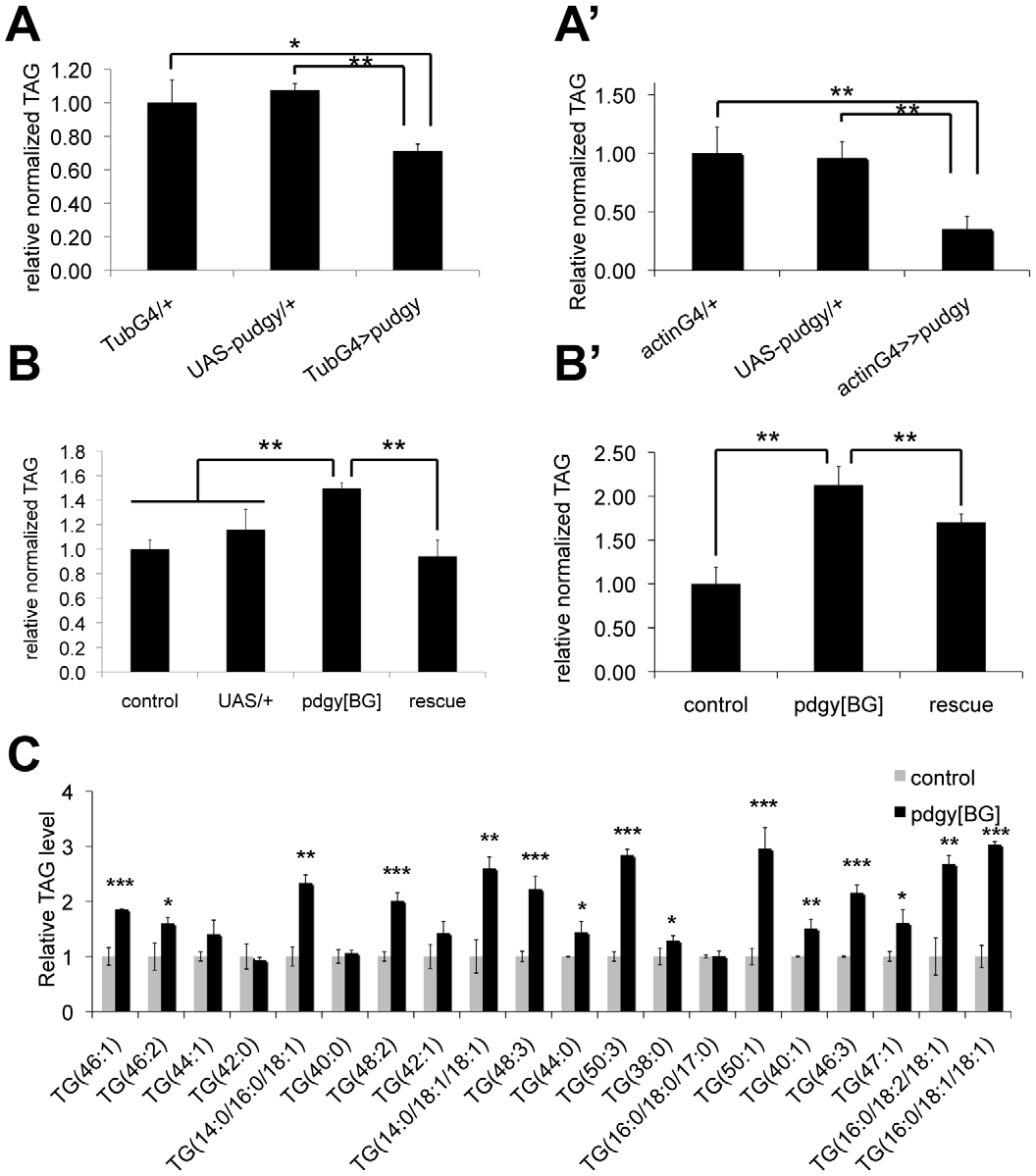
*Pudgy* expression levels regulate organismal lipid homeostasis. (A–A′) *pudgy* overexpression causes leanness. Relative total body triglycerides normalized to total body protein of wL3 larvae (A) or adults (A′) ubiquitously overexpressing *pudgy* from an inducible UAS-transgene with the tubulin-GAL4 or actin-GAL4 drivers (tubG4>>pudgy or actinG4>pudgy), or in the two control parental genotypes, which by themselves do not overexpress *pudgy* (GAL4 only and UAS-pudgy only). (B–B′) *pudgy* mutants are fat. Relative total body triglycerides normalized to total body protein of control or *pdgy[BG]* mutant wL3 larvae (B) or adult males (B′), as well as *pdgy[BG]* mutants simultaneously carrying UAS-pudgy. The *pdgy[BG]* insertion is concurrently a loss-of-function insertion as well as a GAL4 gene trap. (C) Lipidomic profiling of control versus *pdgy[BG]* mutant adult males. Many but not all triacylglyceride (TAG) species are significantly elevated in *pudgy* mutants. The 20 most abundant TAGs are indicated. Values for each TAG are normalized to 1 in control animals. Numbers in parentheses indicate total carbon number of the combined fatty acid chains and total level of desaturation, or values for each individual fatty acid when known. For all panels, Error bars: Std. Dev., *ttest<0.05, **ttest≤0.01, ***ttest<0.001.

The above-mentioned data indicate that insulin/IGF signaling modulates *pudgy* expression in vivo. Therefore, we asked whether modulation of *pudgy* expression has an impact on organismal lipid homeostasis. We first tested the effect of increasing *pudgy* expression. Ubiquitous over-expression of *pudgy* from a transgene using the GAL4/UAS system [31] was sufficient to cause a significant reduction in organismal triglyceride levels both in larvae and in adults (Figure 3A and 3A′ respectively). *pdgy[BG]* homozygous mutants are viable, fertile, and normally patterned (Figure S2A). Conversely to *pudgy* gain-of-function, *pdgy[BG]* mutant larvae and adults have significantly elevated triglyceride levels compared to controls (Figure 3B and 3B′ respectively). This phenotype was fully rescued in larvae and partially rescued in adults by introducing *UAS-pudgy* into the *pdgy[BG]* mutants, since the *pdgy[BG]* insertion is a GAL4 gene trap resulting in both *pudgy* loss-of-function as well as GAL4 expression (Figure 3B and 3B′). A comprehensive lipidomic analysis using Ultra Performance Liquid Chromatography coupled to mass spectrometry (UPLC-MS) of molecular lipid species in *pdgy[BG]* mutant versus control flies revealed that many, but not all, triglyceride species were significantly elevated in *pdgy[BG]* mutant adults (Table S1). The results for the 20 most abundant TAGs are shown in Figure 3C. In addition, levels of some other complex lipids, such as cholesteryl ester (19∶0), were also elevated in *pdgy[BG]* mutants (Table S1). The increased adiposity of *pdgy[BG]* mutants is consistent with the reduced levels of fatty acid oxidation observed in these animals (Figure 2E and 2E′). Furthermore, *Pudgy* mutants do not ingest more than control animals (Figure S3A and S3A′) and have reduced expression of key lipogenic genes such as Acetyl-CoA Carboxylase (ACC) and Fatty Acid Synthase (FAS) (Figure S3B and S3B′), suggesting that mutant animals may be trying to compensate for their increased adiposity. These results are analogous to those observed in ACSL1 knockout mice, which have elevated fat mass [32]. Together, they indicate that the level of expression of ACSs is important for setting steady-state lipid levels both in flies and in mammals.

### Pudgy mutant flies have an altered lipid catabolic profile upon fasting

**Figure 4:**
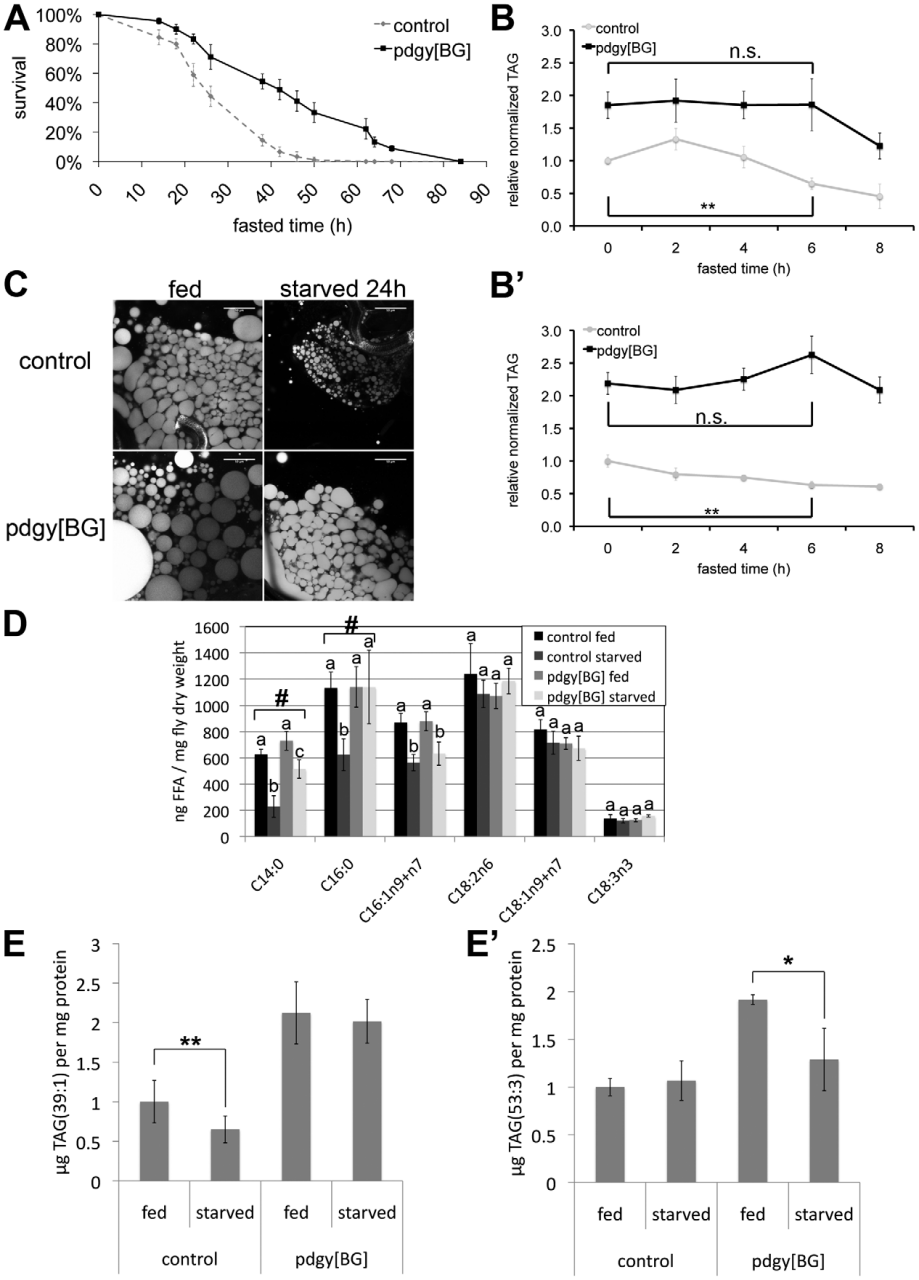
*pudgy* mutants have an altered lipid catabolic profile upon fasting. (A) *pdgy[BG]* mutants have significantly improved survival under starvation conditions. Control w^1118^ (dashed line) and *pdgy[BG]* (solid line) 3-day-old males starved on 0.8% agarose/PBS (n = 90, log rank P = 3×10^−8^). (B–B′) *pdgy[BG]* mutants display delayed lipid catabolism upon starvation. Relative total body triglycerides normalized to total body protein of control and *pdgy[BG]* L2 larvae (84 h AEL) (B) or adults (B′) fasted for 0, 2, 4, 6 or 8 hours. (C) Nile red staining of early L3 larval fat bodies reveals larger lipid droplets in *pdgy[BG]* mutants compared to controls, both under fed conditions and when starved for 24 h on 0.8% agarose/PBS. Scare bars: 50 µm. (D) *Pudgy* mutants have aberrant levels of free fatty acids. Free fatty acid levels obtained by lipidomic profiling of control and *pdgy[BG]* mutant adults under fed and 16-hour fasting (“starved”) conditions. Measurements annotated with different letters are significantly different from each other (ttest<0.01). # indicates lipid species for which the drop in concentration observed in control animals upon fasting is significantly impaired in the mutant. (E–E′) *pdgy[BG]* mutants have an altered profile of lipid catabolism upon fasting. Levels of TAG(39∶1) (E) and TAG(53∶3) (E′) were quantified by UPLC-MS lipidomic profiling of control or *pdgy[BG]* mutant adult males after 0 and 16 hours of fasting. For all panels, assays done in triplicate, Error bars: Std. Dev., * ttest<0.05, ** ttest≤0.01, ***ttest<0.001.

We next studied the physiological consequences of impaired *pudgy* expression in flies upon fasting. Upon complete food withdrawal, *pdgy[BG]* mutants survived significantly longer compared to controls (Figure 4A and Figure S3C). This is likely due in part to the increased adiposity of *pdgy[BG]* mutants, as starvation survival is known to correlate with lipid levels in the fly [25], [33]–[35]. Additionally, this could also be due in part to a reduced rate of lipid catabolism which is nonetheless sufficient to support viability. We therefore tested whether lipid catabolism might also be impaired in *pdgy[BG]* mutants, as they have reduced fatty acid oxidation. Upon food removal, control flies progressively catabolized their triglyceride stores. After 6 hours of fasting, both control larvae and control adult flies significantly reduced their triglyceride stores (Figure 4B and 4B′, grey curves). Control larvae reproducibly displayed an unexpected transient increase in stored triglycerides after 2 hours of fasting before starting to deplete them (Figure 4B). In contrast, *pdgy[BG]* mutants did not show any reduction in triglyceride levels the first 6 hours of starvation (Figure 4B and 4B′, black curves). Only as of 8 hours of starvation did *pdgy[BG]* mutants start depleting their triglycerides stores, completely depleting them by 36 hours of fasting (Figure 4B, 4B′ and Figure S3D), indicating that after an initial period, they were nonetheless able to catabolize lipids. Similar defects could also be observed by staining fat bodies of control and *pdgy[BG]* mutants with Nile Red (Figure 4C). Interestingly, both the extended survival upon food withdrawal as well as the delay in triglyceride consumption the first 6 hours of fasting are also observed in mutants for another gene involved in lipid catabolism - the fly homolog of adipocyte triglyceride lipase, brummer [24], [36].

To study lipid catabolism in *pdgy[BG]* mutants in more detail, we performed quantitative lipidomic profiling of fed versus fasting flies. Since the direct substrates of ACS action are free fatty acids, we first quantified free fatty acids in *pdgy[BG]* mutant and control animals (Figure 4D). Upon starvation, levels of free C14:0, C16:0 and C16:1 drop in control animals (Figure 4D). Since levels of free fatty acids reflect the balance between fatty acid lipolysis and fatty acid ligation to CoA (Figure 1A), this indicates that upon starvation ACSs become activated in order to handle the increased production of free fatty acids coming from triglyceride lipolysis. In contrast, in *pdgy[BG]* mutants, levels of free C14:0 and C16:0 remained aberrantly high (Figure 4D), as expected from impaired ACS activity in the *pudgy* mutants. Defects were only apparent in a subset of free fatty acids (Figure 4D) suggesting that the metabolism of all fatty acids might not be affected equally by loss of pudgy in vivo.

We next performed quantitative lipidomic profiling to detect all TAG species in fed versus fasting control and *pdgy[BG]* flies. Although many TAG species are normally catabolized in *pdgy[BG]* mutants (Table S2), some species are not. For instance, levels of TAG(39∶1) dropped in control animals upon fasting but remained elevated in *pdgy[BG]* mutants (Figure 4E), whereas TAG(53∶3) remained constant in control animals but dropped in *pdgy[BG]* mutants (Figure 4E′). Therefore, *pudgy* mutants display an altered profile in the catabolism of lipid species. Consistent with this, *pdgy[BG]* mutants have aberrant expression of a large number of putative lipases, elongases and ACSs (Figure S4) suggesting that lipid catabolic pathways may be readjusting in response to loss of *pudgy*. In sum, our data indicate that *pudgy* mutants are initially defective in the catabolism of fatty acids, but after an initial period are able to catabolize all triglycerides, albeit with a different pattern compared to controls.

### Pudgy mutants also display insulin signaling and carbohydrate metabolism phenotypes

**Figure 5:**
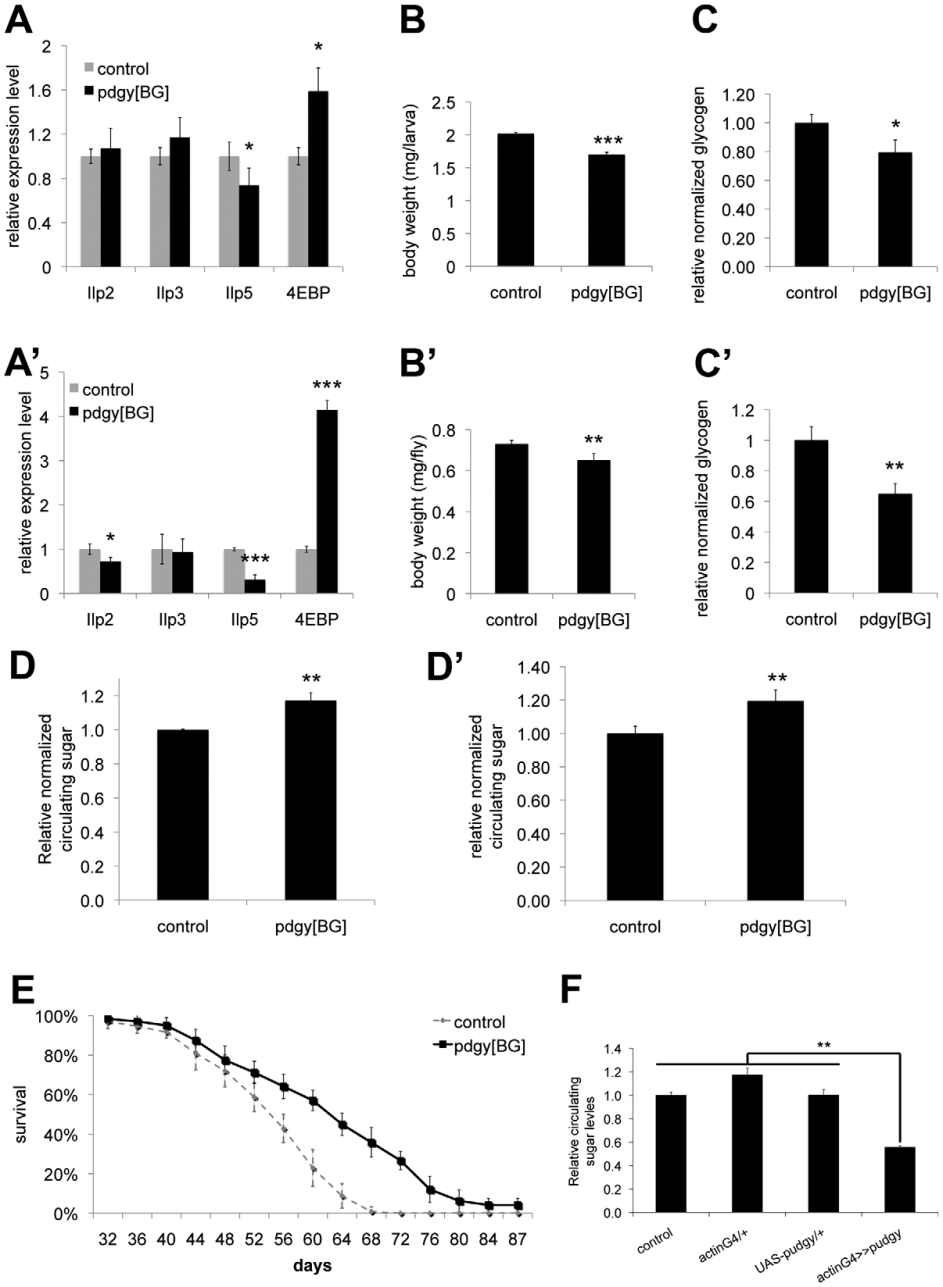
Pudgy mutants have reduced insulin signaling and carbohydrate metabolism defects. (A–A′) *Pudgy* mutant feeding L3 larvae (96 h AEL) (A) and adults (A′) have reduced expression of ILPs, and elevated expression of 4E-BP, a gene suppressed by insulin signaling. Assayed by quantitative RT-PCR relative to rp49. (B–B′) *Pudgy* mutants are mildly reduced in size. Weight of control (w^1118^) and *pdgy[BG]* wL3 larvae (B) or adult males (B′). (C–C′) *Pudgy* mutants have reduced glycogen stores. Total body glycogen normalized to total body weight for wL3 larvae (C) or adults (C′). (D–D′) *Pudgy* mutants have elevated levels of circulating sugars. Relative trehalose levels of control and *pdgy[BG]* mutant wandering 3rd instar larvae (D) or adults (D′). (E) *pdgy[BG]* mutants have extended lifespan. Lifespan of control (dotted line) or *pdgy[BG]* mutant (black line) males, reared under controlled growth conditions and maintained on normal laboratory food (30 flies per tube, in octuplicate, log rank P = 10^−15^). (F) *Pudgy* overexpression causes hypoglycemia. Relative trehalose levels normalized to total body weight of wandering 3rd instar larvae ubiquitously overexpressing pudgy from an inducible UAS-transgene with the actin-GAL4 driver (actinG4>>pudgy) or in the two control parental genotypes, which by themselves do not overexpress pudgy (actinG4/+ and UAS-pudgy/+). For all panels unless noted, assays done in triplicate, Error bars: Std. Dev., *ttest<0.05, **ttest≤0.01, ***ttest≤0.001.

Interestingly, although Pudgy is an enzyme involved in lipid metabolism, we found that *pudgy* mutants also have a number of other non-lipid phenotypes. *Pudgy* mutants had significantly reduced expression of insulin-like peptides (Figure 5A and 5A′). Correspondingly, they had elevated expression of 4E-BP, a direct FOXO target, consistent with reduced insulin signaling in these animals (Figure 5A and 5A′). *Pudgy* mutants also have two phenotypes associated with reduced insulin signaling: they are mildly, but significantly reduced in size compared to controls (Figure 5B and 5B′) and they are long-lived (Figure 5E). In addition, *pudgy* mutants also have reduced glycogen stores (Figure 5C and 5C′) and increased circulating sugars (Figure 5D and 5D′) suggesting elevated mobilization of carbohydrates. Conversely, pudgy overexpression leads to reduced circulating sugars (Figure 5F). Although these phenotypes are not the focus of this story, and we do not know their underlying molecular mechanisms, they are worth noting as they probably represent crosstalk mechanisms in *pdgy[BG]* animals caused by their elevated lipid stores and reduced lipid oxidation, of interest for future studies.

### Expression of ACSs is also regulated by insulin signaling in mammals

**Figure 6:**
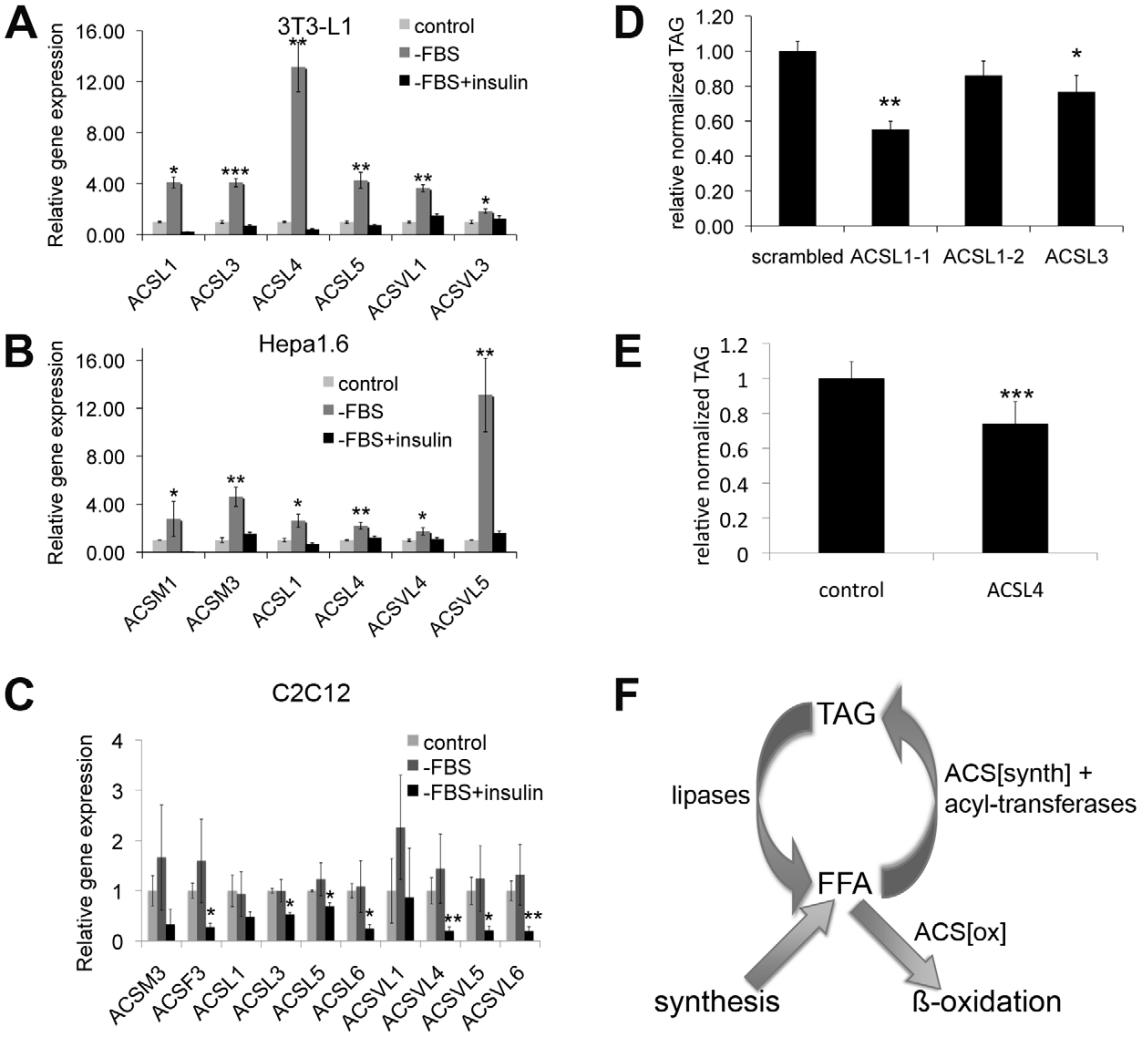
Expression of many ACSs is regulated by insulin in mammals, and their expression level affects lipid homeostasis. (A–C) Expression of selected Acyl-CoA synthetases in 3T3-L1 adipocytes (A), Hepa1.6 hepatoma cell line (B) or C2–C12 myotubes (C) treated with complete DMEM (control), DMEM lacking serum (−FBS), or DMEM lacking serum but supplemented with 5 µg/mL insulin (−FBS+insulin). Cells were serum starved for 1 hour (3T3-L1) or for 4 hours (Hepa1.6 and C2C12), and then treated with or without insulin (5 µg/ml for 3T3-L1 and Hepa1.6, 100 nM for C2C12) for 1 (3T3-L1 and Hepa1.6) or 2 hours (C2C12). Expression was measured by quantitative RT-PCR normalized to beta-actin. Since C2–C12 myotubes are differentiated by culturing in medium with low serum, the control and –FBS conditions are similar. Stars indicate statistical significance relative to control (3T3-L1, Hepa1.6) or to –FBS (C2C12). (D) Knockdown of ACSL1 or ACSL3 expression causes reduced triglyceride levels in differentiated 3T3-L1s. Relative total triglycerides normalized to total protein for 3T3-L1 cells treated with control siRNA (scambled) or siRNA targeting ACSL1 (ACSL1-1 and ACSL1-2) or ACSL3 shortly prior to differentiation. (E) Knockdown of ACSL4 expression causes reduced triglyceride levels in differentiated 3T3-L1 cells. Relative total triglycerides normalized to total adiponectin levels, a marker for differentiation, for control 3T3-L1s or 3T3-L1s treated with shRNA targeting ACSL4. (F) Simplified schematic representation of fatty acid metabolism. Fatty acids cycle between a free form (FFA) and a stored form as triacylglycerol (TAG). FFA are released from TAG by the action of lipases, wherease FFA are re-esterified via the sequential action of a subset of acyl-CoA synthetases and acyl-transferases. Neither lipases nor acyl-transferases can create or destroy fatty acids. Fatty acids are destroyed via the action of acyl-CoA synthetases which activate them for beta-oxidation. For all panels, assays done in triplicate. Error bars: std. dev. * ttest<0.05, **ttest<0.01, ***ttest<0.001.

We next asked whether our two central observations from Drosophila—that insulin signaling regulates ACS expression and that ACS expression levels are important for lipid homeostasis—can also be observed in a mammalian context. To this end, we treated three different cell types, 3T3-L1 adipocytes, Hepa1.6 hepatocytes and C2C12 myotubes, representing three different tissues of metabolic importance, in the presence or absence of insulin, and measured by quantitative RT-PCR the expression of all medium-chain, long-chain and very-long-chain ACSs. Reported in Figure 6 are the ACSs who's transcription was regulated in a manner similar to that of *pudgy*, i.e. repressed by insulin. In addition, other ACSs were either not transcriptionally regulated by insulin, or were induced by insulin (Table S3A and S3B). In 3T3-L1 adipocytes, expression of six different ACSs was up-regulated upon removal of serum (Figure 6A). This up-regulation was suppressed if insulin was supplied upon serum removal, indicating that the up-regulation was specific for insulin signaling (Figure 6A). In particular, expression of ACSL4 increased very strongly, 12-fold, within the short 1-hour time window of serum removal (Figure 6A). Likewise, expression of 6 different ACSs increased in an insulin-dependent manner in Hepa1.6 hepatocytes upon serum removal, with ACSVL5 increasing 13-fold (Figure 6B). Although some ACSs are similarly regulated by insulin in both cell types, such as ACSL1, other ACSs are specifically regulated in one cell type or the other, probably reflecting the specific function of each tissue. Finally, a number of ACSs were also regulated by insulin in C2–C12 myotubes (Figure 6C). (Since C2–C12 myoblasts are differentiated into myotubes by culturing in low-serum conditions, the ‘control’ and ‘serum-deprived’ conditions are similar in gene expression.)

To test whether the level of expression of ACSs in 3T3-L1 adipocytes affects lipid homeostasis, we knocked down expression of three different ACSs – ACSL1, ACSL3 and ACSL4. Knockdown of ACSL1 and ACSL3 using siRNAs caused reduced triglyceride storage in differentiated 3T3-L1s (Figure 6D) in a manner that correlated with relative knock-down efficiency (Figure S5A, S5B). This is consistent with previous reports that ACSL1 promotes fatty acid uptake and incorporation into TAG in 3T3-L1s [37], [38]. Using a different approach, 3T3-L1s expressing an shRNA targeting ACSL4 also had reduced triglyceride storage (Figure 6E and Figure S5C).

## Discussion

### ACS expression regulates organismal lipid homeostasis

Fatty acid (FA) catabolism represents an important energy yielding mechanism for cells and organisms, contributing up to 50–60% of a person's energy expenditure under aerobic exercise conditions [39]. Fatty acid catabolism can be envisioned in two steps (Figure 6F). First, fatty acids are mobilized from stored triacylglycerols (TAG) via the activity of lipases to yield free fatty acids. Second, the free fatty acids are oxidized, yielding energy. Traditionally, textbook knowledge considers the first step – mobilization via lipases – to be the important regulated step. However, several lines of reasoning suggest that lipolysis cannot be the only important regulated event in the fatty acid catabolic pathway. Firstly, liberation of free fatty acids from TAG does not necessarily channel them towards beta-oxidation. Free fatty acids can have several fates, including not only beta-oxidation but also fatty acid elongation (yielding very long chain fatty acids) and re-esterification to generate complex lipids including TAG [40], [41]. In fact, a large fraction of FAs liberated from TAG participate in a ‘futile’ cycle, being re-esterified to generate new TAG [42]. Quantitative estimates of the triglyceride/fatty acid cycle in humans and in animals show that only a small fraction of the FFA released as a result of lipolysis in adipose tissue are oxidized, and the majority are re-esterified to triglycerides in various tissues [43]. Secondly, elevated levels of free FA are believed to be deleterious to animals, causing lipotoxicity and contributing towards insulin resistance [40]. Therefore, increased FA levels due to increased lipolysis without concurrent upregulation of downstream biochemical pathways might actually be noxious to the animal. We identify here the subsequent step in fatty acid catabolism - coupling of fatty acids to CoA via ACSs - as an additional, important regulated step in lipid catabolism. A priori, it was possible that the level of expression of *pudgy in vivo* was not limiting for lipid oxidation, and that lipid catabolism in Drosophila is only regulated by availability of free fatty acids via lipolysis. However, our data suggest this is not the case. Both a reduction and an increase in *pudgy* levels effects total lipid levels in the fly (Figures 3A, 3A′, 3B, 3B′), indicating that regulation of *pudgy* levels contributes significantly to total body lipid homeostasis. This makes sense in light of the fact that free fatty acids can have multiple different fates once released from triglycerides, such as beta-oxidation or re-esterification to form triglycerides. Therefore the relative activities of biochemical reactions downstream of lipolysis are important for determining the fate of the released fatty acids. In particular, the balance in expression and activity of ACSs that activate fatty acids for beta-oxidation versus lipid biosynthesis may be of particular importance. In Drosophila, upon starvation, FOXO upregulates expression of the fly adipocyte triglyceride lipase homolog, brummer [24]. By upregulating expression of both brummer and pudgy, FOXO mounts a concerted effort towards channeling fatty acids from their stored form towards beta-oxidation.

It may appear surprising that blocking fatty acid ß-oxidation via mutation of *pudgy* leads to increased TAG levels in the animal, since lipolysis is often considered to be the key step in regulating TAG levels. Indeed, via the actions of lipases and acyl-transferases, fatty acids cycle between a free form and a stored TAG form (Figure 6F), however neither of these enzymatic activities either creates or destroys fatty acids. The steady-state level of fatty acids in an organism depends only on the relative balance of fatty acid synthesis/uptake versus fatty acid oxidation. Therefore, reducing ß-oxidation increases total organismal fatty acids. Since free fatty acids are in equilibrium with the stored TAG form, this entails an increase in TAG levels (Figure 6F).

An alternate interpretation of our data is that the observed delay in TAG consumption reflects a reduced global metabolic rate caused indirectly by lack of pudgy activity. We believe this interpretation is unlikely, because a global redution in metabolic rate would be expected to lead to a concomitant increase in the levels of both stored lipids and stored carbohydrates (ie glycogen). Pudgy mutants, however, have elevated lipids levels but reduced glycogen levels, suggesting a lipid-specific defect in accordance with pudgy's ACS function.

### Insulin/IGF signaling regulates lipid homeostasis in part via ACS expression

Insulin/IGF signaling is known to control lipid biosynthesis in part via SREBP1, and lipid catabolism via regulation of lipases such as hormone sensitive lipase and via decreasing the rate of fatty acid entry into mitochondria [15], [44], [45]. We identify here the ACS *CG9009/pudgy* as one molecular link between the insulin signaling pathway and lipid catabolism in Drosophila. We find that *pudgy* is a transcriptional target gene of the insulin pathway which is directly regulated by FOXO. By repressing *pudgy* expression, insulin blocks the channeling of fatty acids towards the beta-oxidative pathway. Insulin has been reported to induce expression of two ACSs in mammals - ACSL5 via a mechanism involving SREBP1c [46], and ACSL6 via an unknown mechanism [47] – however to our knowledge pudgy is the first example of an ACS which is repressed by insulin. Likewise, although pudgy belongs to a clade of ACSs that does not also include human paralogs, we identify a number of human ACSs that are transcriptionally repressed by insulin in mammalian cells, analogously to pudgy.

### Pudgy mutants have altered metabolic parameters

We find that *pudgy* mutants have a significant number of metabolic alterations. For instance, in addition to the changes in lipid metabolism, we find that *pudgy* mutants have reduced glycogen stores and increased circulating sugars. Although the underlying mechanism is unclear, one plausible explanation is that *pudgy* mutants need to rely more on glucose mobilization to maintain cellular energy levels, to compensate for reduced fatty acid beta-oxidation, which is normally a significant energy source.

We also find that *pudgy* mutants have a different profile of lipid homeostasis and starvation-induced catabolism compared to controls. Under fed conditions, some lipid species in *pudgy* mutants are highly elevated, such as TAG(50∶1) which is almost 3-fold the normal levels, whereas others such as TAG(42∶0) are unperturbed (Figure 3C). Likewise, during starvation, the catabolism of lipid species is altered, with some TAGs being catabolized more readily and some less readily compared to controls (Figure 4E and 4E′). Fatty acid species are linked to each other via a complex network of biochemical pathways involving saturases, desaturases, elongases, ACSs, lipases, etc. This ‘landscape’ of lipid species is clearly perturbed by removal of *pudgy*. This perturbation might be partly a direct consequence of loss of pudgy, and partly an attempt of the system to compensate. Indeed, at the gene expression level, a very large proportion of genes with putative functions in fatty acid metabolism are altered in *pudgy* mutants, suggestive of compensatory mechanisms (Figure S4). For instance, the elongase eloF is more than 2-fold up-regulated in the *pudgy* mutant, and the ACS CG6432 is dramatically down-regulated.

In sum, we identify here the ACS pudgy as a transcriptional target of insulin signaling, and show that modulation of pudgy expression levels causes changes in steady-state lipid levels in the fly. Mammalian tissue culture experiments suggest similar mechanism may be at work in mammalian cells.

## Materials and Methods

### Constructs and fly strains

A list of oligos used for clonings and quantitative PCRs can be found in Supplemental Materials & Methods (Text S1). Additional oligos sequences are available upon request. UAS-pudgy was generated by cloning the CG9009 coding sequence, obtained by RT-PCR as an XhoI-XbaI fragment, into the XhoI-XbaI sites of pUAST. The mito-GFP ORF, encoding the 31 amino acid mitochondrial import sequence from human cytochrome C oxidase subunit VIII fused to the N terminus of GFP, was amplified from flies carrying mito-GFP (Bloomington Stock Center, [48]) and cloned into pCasper4 carrying a tubulin promoter. For luciferase assays, the FOXO enhancer region of *pudgy* intron 1 was amplified as a KpnI-KpnI fragment and cloned into the KpnI site of a luciferase plasmid containing the Adh basal promoter, described in [20]. Remaining constructs for the FOXO luciferase assay were described previously [20]. FOXO^21^ and FOXO^25^ flies [23]; P{GT1}BG02662 flies and actin-GAL4 flies (Bloomington Stock Center).

### Controlled growth conditions for metabolic and longevity analyses

For all metabolic, respiratory, and longevity analyses, animals were reared under strictly controlled growth conditions. Eggs were collected on apple plates, and newly hatched L1 larvae were seeded in vials at a density of 60/vial and grown at 25°C without yeast supplementation. Adult flies were then aged 3 days for analysis. All assays were done in triplicate. Our fly food recipe is as previously reported [49]. Metabolic, starvation and longevity assays were performed as in [40] and as detailed in Text S1 (Supplemental Materials and Methods).

### Lipidomics analysis

Growth controlled w^1118^ and *pdgy[BG]* mutant males were aged 3 days, and then fed normal food or starved on 0.8% agarose/PBS overnight (16 hours). The flies were frozen in liquid nitrogen and cryo-dried. The samples were then analyzed by UPLC-QTof-MS using an Acquity BEH C_18_ (1.7 µm 2.1×100 mm) column and electrospray ionization in positive ion mode. Details are provided in the Supplemental Materials & Methods (Text S1).

### In vitro ACS activity assay

His-tagged pudgy protein was obtained by cloning the coding sequence into pET23d, expressing it in BL21 E. coli, and purifying it using Ni-NTA Agarose beads (Qiagen). 4.2 µg of recombinant pudgy-His, or an equivalent amount of eluate from a parallel purification using bacteria not expressing pudgy-His (circa 4.3 mg), were added into reaction buffer (50 mM Tris–HCl pH 7.8, 10 mM sodium acetate, 4 mM ATP, 0.15 mM CoA, 1 mM magnesium chloride, 10 mM DTT) with 10 nmol free fatty acid. After incubated at 37C for 30 min, the synthesized acyl-CoA was detected using the Free Fatty Acids Quantification Kit (Biovision), omitting the ACS incubation step. As a positive control, 4.2 µg of ACS supplied with the kit was used.

### Oxygen consumption measurements

Growth-controlled, wandering third instar larvae were cleaned in cold PBS, dried on filter paper and weighed. Larvae were then dissected into ice-cold BIOS buffer (2.77 mM CaK_2_EGTA, 7.23 mM K_2_EGTA, 5.77 mM Na_2_ATP, 6.56 mM MgCl_2_·6H_2_O, 20 mM Taurine, 15 mM Na_2_Phospho-creatine, 20 mM Imidazole, 0.5 mM DTT, 50 mM MES) and subsequently permeabilized with 4 mM digitonin in BIOS buffer for 15 min at 4°C in a shaker. Tissues were then resuspended in ice-cold FAO medium (110 mM NaCl, 4.7 mM KCl, 2 mM MgSO_4_, 1.2 mM Na_2_HPO_4_, 2.5 mM glucose adjusted to pH 7.4, supplemented with 0.5 mM carnitine). Oxygen consumption was measured using a Clark electrode and normalized to animal body weight. Etomoxir was added (50 µM or 300 µM) to block acyl-CoA transport via CPTI.

### Detailed procedures

Detailed procedures of methods used are included in the Supplemental Materials & Methods (Text S1).

## Supporting Information

Figure S1 Pudgy overexpression is sufficient to increase lipid beta-oxidation rates. (A) CPTI-dependent O_2_ consumption, calculated by subtracting the rate of oxygen consumption in the presence of 300 µM etomoxir (ie CPTI independent) from the total rate of oxygen consumption in the absence of drug, is indicated for larval tissues from three genotypes: two parental genotypes which do not overexpress pudgy (Tubulin-GAL4/+. and UAS-pudgy/+) and the experimental genotype which ubiquitously expresses Pudgy (Tubulin-GAL4/UAS-Pudgy).
(TIF)

Figure S2 *pdgy[BG]* mutants are normally developed and fatter in all development stages. (A) Image of growth-controlled, 3 day old, *pudgy* mutant and control male flies. Mutant flies have no detectable patterning defects. (B) *pudgy* mutants are fat at all stages of development. Relative total body triglycerides normalized to total body protein of control or *pdgy[BG]* mutant males at wandering 3^rd^ instar stage (wL3), or at 2, 4 and 10 days after adult eclosion (D2, D4 and D10 respectively). For all panels, assays done in triplicate. Error bars: Std. Dev. ***ttest<0.001.
(TIF)

Figure S3 Pudgy mutants do not eat more than controls, and have reduced expression of lipogenic genes. (A–A′) Food intake of *pdgy[BG]* mutants is not elevated compared to controls, both in larvae (96 h AEL) (A) and in 3-day old adults (A′). Food was supplemented with 0.5% Blue 9 dye, and ingested food per animal was quantified. n = 6, done in triplicate. (B–B′) *pdgy[BG]* mutant 3^rd^ instar larvae (96 h AEL) (B) and adults (B′) do not have elevated expression of acetyl-CoA carboxylase (dACC), or Fatty Acid Synthase (dFAS, CG3523). Assayed by quantitative RT-PCR relative to rp49. (C) *pdgy[BG]* mutants have significantly improved survival under starvation conditions. Control w^1118^ (dashed line) and *pdgy[BG]* (solid line) L2 larvae (72 h after egg laying) were starved on 0.8% agarose/PBS (n = 90, log rank P = 9×10^−8^). (D) Relative total body triglycerides normalized to total body protein of control and *pdgy[BG]* adult males, fasted for 0, 12, 24 or 36 hours. In all panels, *ttest<0.05, **ttest<0.01, ***ttest<0.001.
(TIF)

Figure S4 Expression of lipases, elongases, desaturases and ACSs in control and pudgy mutants upon feeding and fasting. Expression of multiple putative lipases (A), elongases and desaturases (B) and ACSs (C) is significantly altered in *pdgy[BG]* mutants both under fed and fasted conditions. Data are from L3 larvae (96 hours AEL), starved on 0.8% agarose/PBS for 4 hours. Gene expression was measured by quantitative RT-PCR relative to rp49. Genes in (A) and (B) were selected based on Flybase Gene Ontology annotations, and genes in (C) are the complete set of ACSs identified in [27]. In all panels, *ttest<0.05, **ttest<0.01, ***ttest<0.001.
(TIF)

Figure S5 Knockdown efficiency of siRNAs for mouse ACSs. Knock-down efficiency in differentiated 3T3-L1s treated with siRNAs targeting ASCL1 (A) and ACSL3 (B) shortly prior to differentiation or with shRNA targeting ACSL4 (C). Targeted genes were analyzed by Q-PCR normalized to ß-actin. For all panels, assays done in triplicates. Error bars: Std. Dev., *ttest<0.05, **ttest<0.01, ***ttest<0.001.
(TIF)

Table S1 Lipidomic profile of pudgy[BG] mutant and control animals. Lipid levels, in mg lipid per mg protein, in 3-day old control and *pudgy[BG]* mutant males. Average values and standard deviation for triplicate biological replicates are indicated, as well as the student t-test p-value indicating significance of the difference between controls and mutants. Values in parenthesis in the lipid names indicate the total number of carbons in the fatty acids chains, and the total level or desaturation. Ceramide (Cer), Cholesterol ester (ChoE), Diacylglycerol (DG), Lyso-Phosphatidylcholine (LysoPC), Monoacylglycerol (MG), Phosphatidic Acid (PA), Phosphatidylcholine (PC), Phosphatidylethanolamine (PE), Phosphatidylglycerol (PG), Phosphatidylserine (PS), Sphingomyelin (SM), Triacylglycerol (TG).
(PDF)

Table S2 TAG catabolism in pudgy[BG] mutant and control animals after 16 hours starvation. Lipid levels, in mg lipid per mg protein, in 3-day old control and pudgy[BG] mutant males starved for 0 or 16 hours. Average values and standard deviation for triplicate biological replicates are indicated. Values in parenthesis in the lipid names indicate the total number of carbons in the fatty acids chains, and the total level or desaturation. Ceramide (Cer), Cholesterol ester (ChoE), Diacylglycerol (DG), Lyso- Phosphatidylcholine (LysoPC), Monoacylglycerol (MG), Phosphatidic Acid (PA), Phosphatidylcholine (PC), Phosphatidylethanolamine (PE), Phosphatidylglycerol (PG), Phosphatidylserine (PS), Sphingomyelin (SM), Triacylglycerol (TG).
(PDF)

Table S3 Expression of mouse ACSs in Hepa1.6 and 3T3-L1 cells in response to serum removal (“−FBS”), or serum removal supplemented with insulin (“−FBS+insulin”) as in Figure 6 of the main text.
(PDF)

Text S1 Supplemental Materials and Methods.
(DOC)
