# Supplementary figures and images for: Insulin Signaling Regulates Fatty Acid Catabolism at the Level of CoA Activation
Source: PLoS Genet. 2012 Jan 19;8(1):e1002478. doi: 10.1371/journal.pgen.1002478 (PMC3261918; doi:10.1371/journal.pgen.1002478)

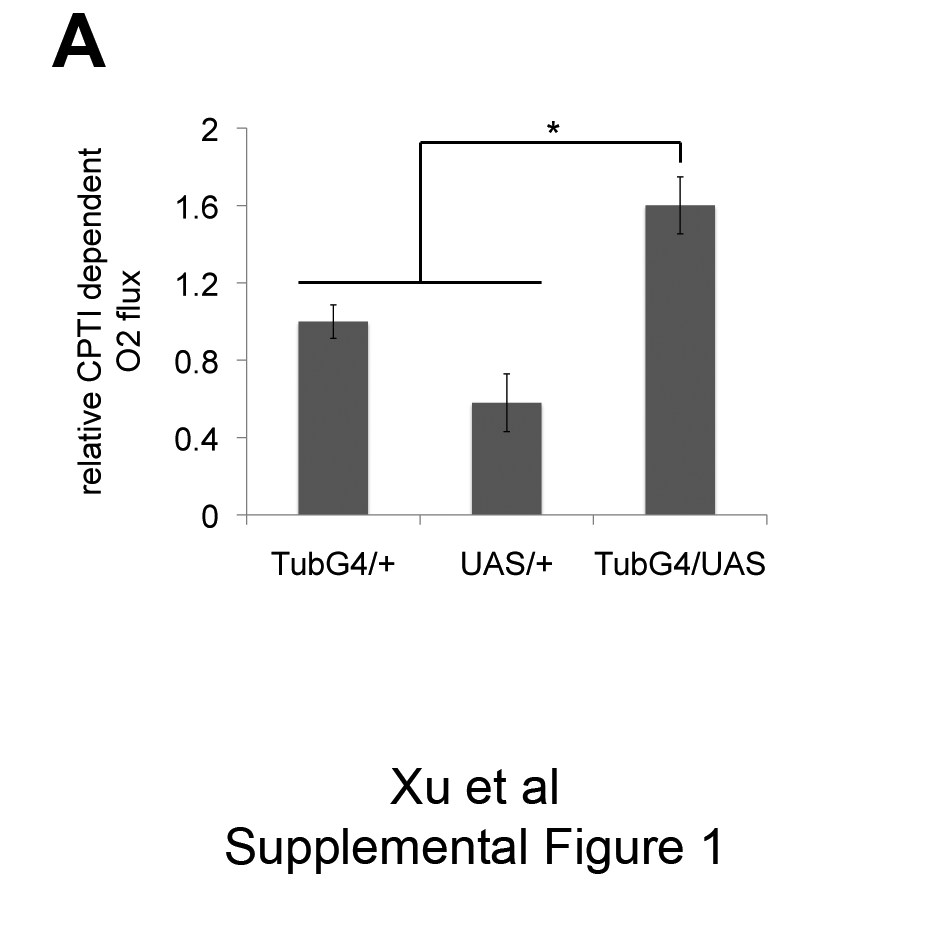

Supplement: Figure S1 — Pudgy overexpression is sufficient to increase lipid beta-oxidation rates. (A) CPTI-dependent O2 consumption, calculated by subtracting the rate of oxygen consumption in the presence of 300 µM etomoxir (ie CPTI independent) from the total rate of oxygen consumption in the absence of drug, is indicated for larval tissues from three genotypes: two parental genotypes which do not overexpress pudgy (Tubulin-GAL4/+. and UAS-pudgy/+) and the experimental genotype which ubiquitously expresses Pudgy (Tubulin-GAL4/UAS-Pudgy). (TIF) [file pgen.1002478.s001.tif]

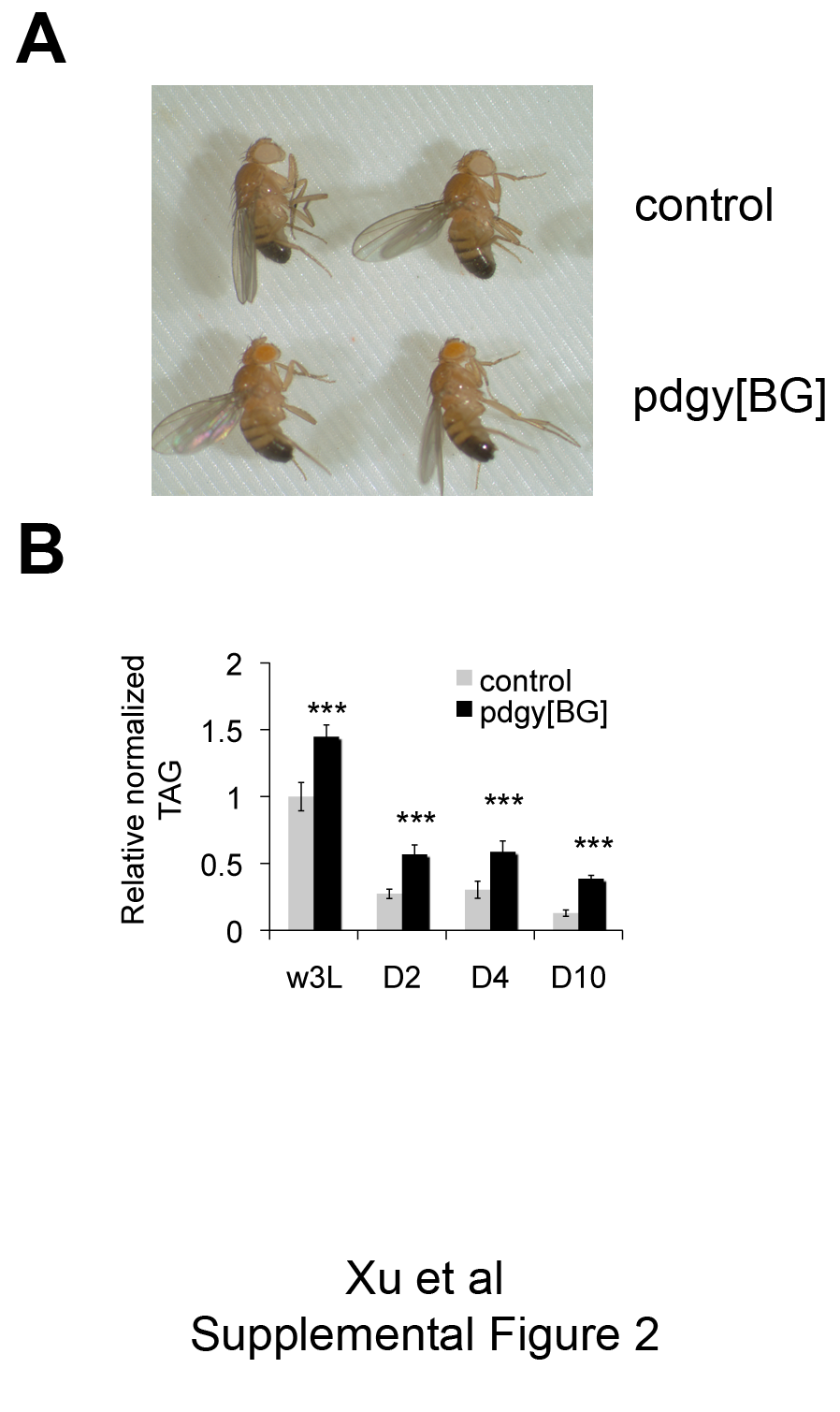

Supplement: Figure S2 — pdgy[BG] mutants are normally developed and fatter in all development stages. (A) Image of growth-controlled, 3 day old, pudgy mutant and control male flies. Mutant flies have no detectable patterning defects. (B) pudgy mutants are fat at all stages of development. Relative total body triglycerides normalized to total body protein of control or pdgy[BG] mutant males at wandering 3rd instar stage (wL3), or at 2, 4 and 10 days after adult eclosion (D2, D4 and D10 respectively). For all panels, assays done in triplicate. Error bars: Std. Dev. ***ttest<0.001. (TIF) [file pgen.1002478.s002.tif]

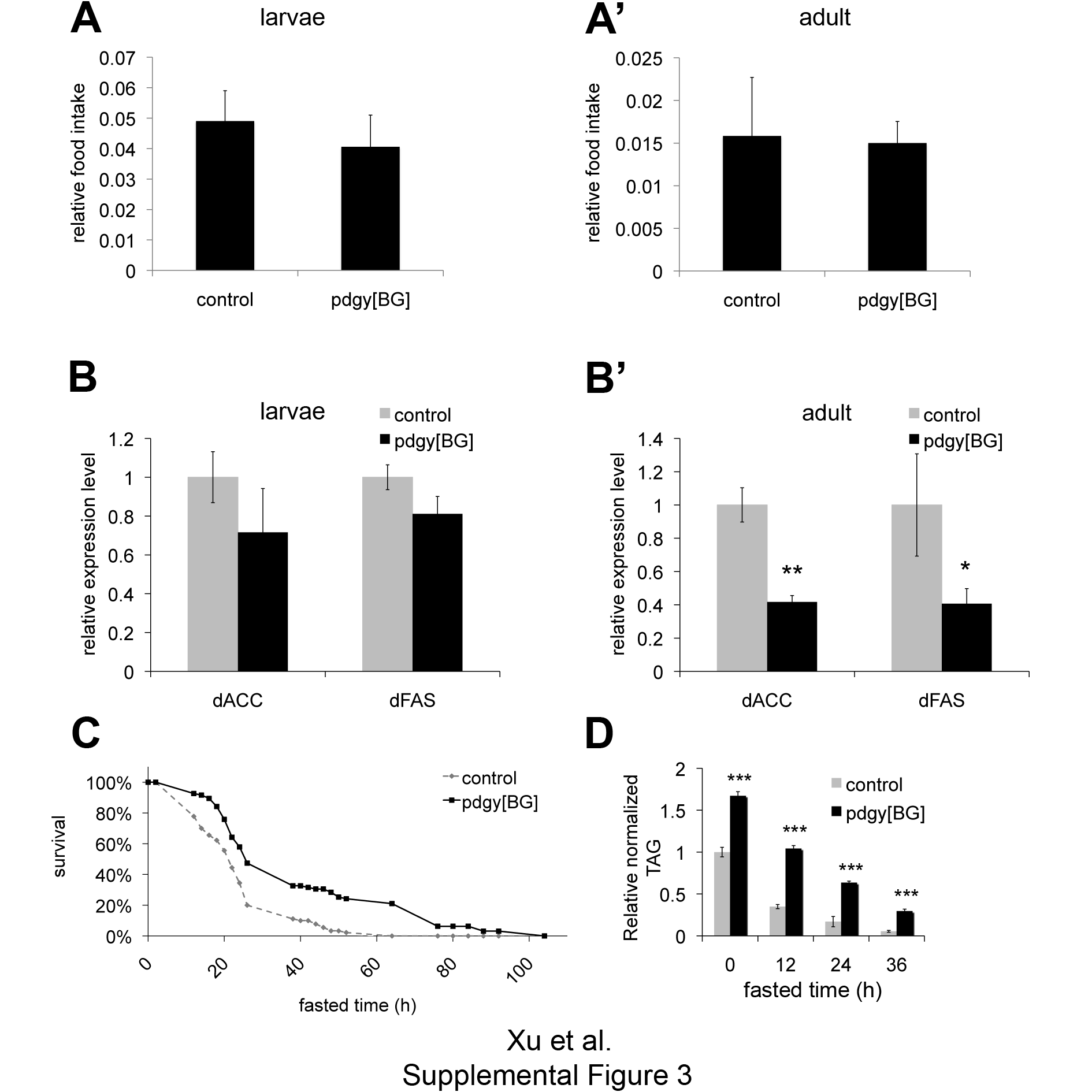

Supplement: Figure S3 — Pudgy mutants do not eat more than controls, and have reduced expression of lipogenic genes. (A–A′) Food intake of pdgy[BG] mutants is not elevated compared to controls, both in larvae (96 h AEL) (A) and in 3-day old adults (A′). Food was supplemented with 0.5% Blue 9 dye, and ingested food per animal was quantified. n = 6, done in triplicate. (B–B′) pdgy[BG] mutant 3rd instar larvae (96 h AEL) (B) and adults (B′) do not have elevated expression of acetyl-CoA carboxylase (dACC), or Fatty Acid Synthase (dFAS, CG3523). Assayed by quantitative RT-PCR relative to rp49. (C) pdgy[BG] mutants have significantly improved survival under starvation conditions. Control w1118 (dashed line) and pdgy[BG] (solid line) L2 larvae (72 h after egg laying) were starved on 0.8% agarose/PBS (n = 90, log rank P = 9×10^−8^). (D) Relative total body triglycerides normalized to total body protein of control and pdgy[BG] adult males, fasted for 0, 12, 24 or 36 hours. In all panels, *ttest<0.05, **ttest<0.01, ***ttest<0.001. (TIF) [file pgen.1002478.s003.tif]

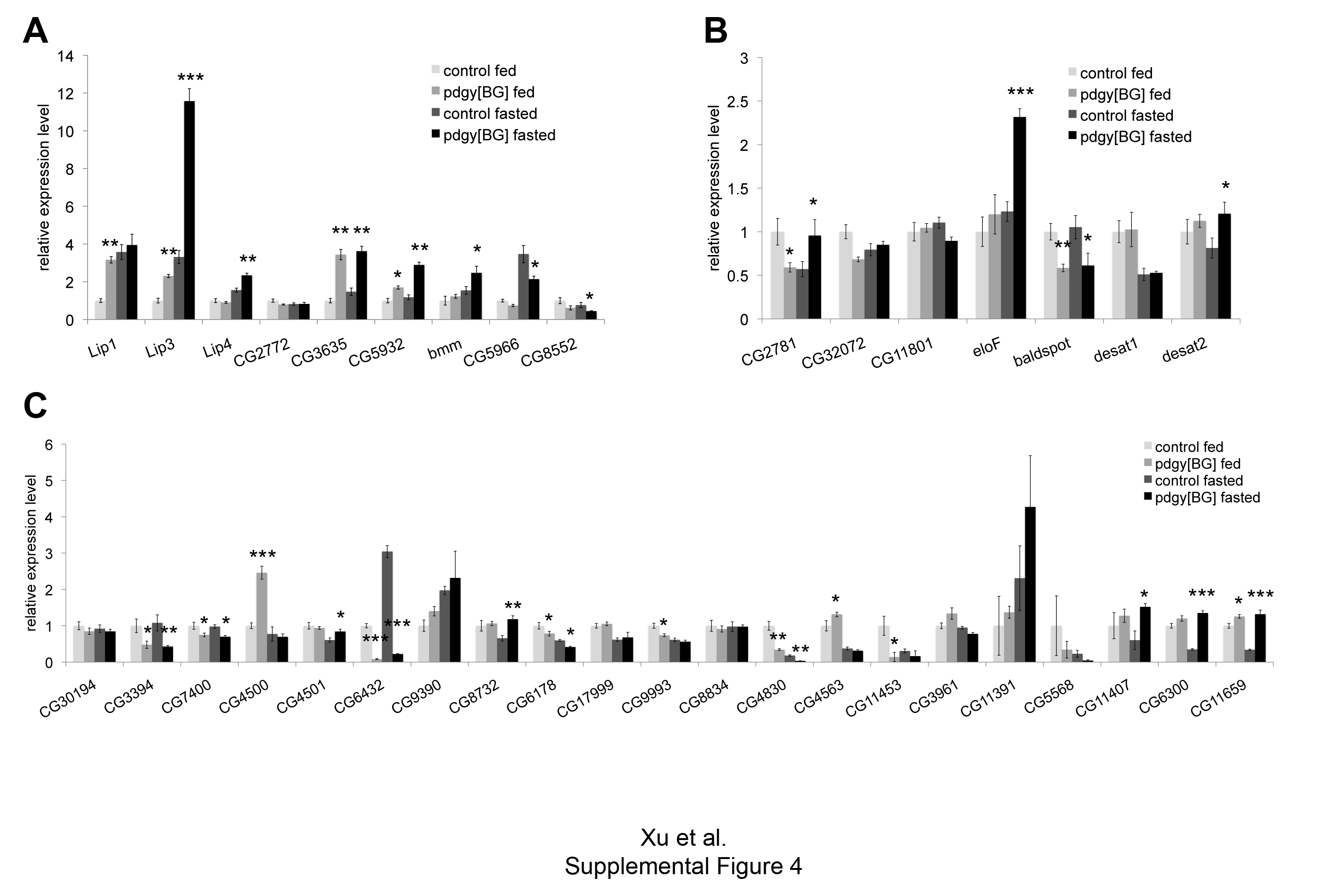

Supplement: Figure S4 — Expression of lipases, elongases, desaturases and ACSs in control and pudgy mutants upon feeding and fasting. Expression of multiple putative lipases (A), elongases and desaturases (B) and ACSs (C) is significantly altered in pdgy[BG] mutants both under fed and fasted conditions. Data are from L3 larvae (96 hours AEL), starved on 0.8% agarose/PBS for 4 hours. Gene expression was measured by quantitative RT-PCR relative to rp49. Genes in (A) and (B) were selected based on Flybase Gene Ontology annotations, and genes in (C) are the complete set of ACSs identified in [27]. In all panels, *ttest<0.05, **ttest<0.01, ***ttest<0.001. (TIF) [file pgen.1002478.s004.tif]

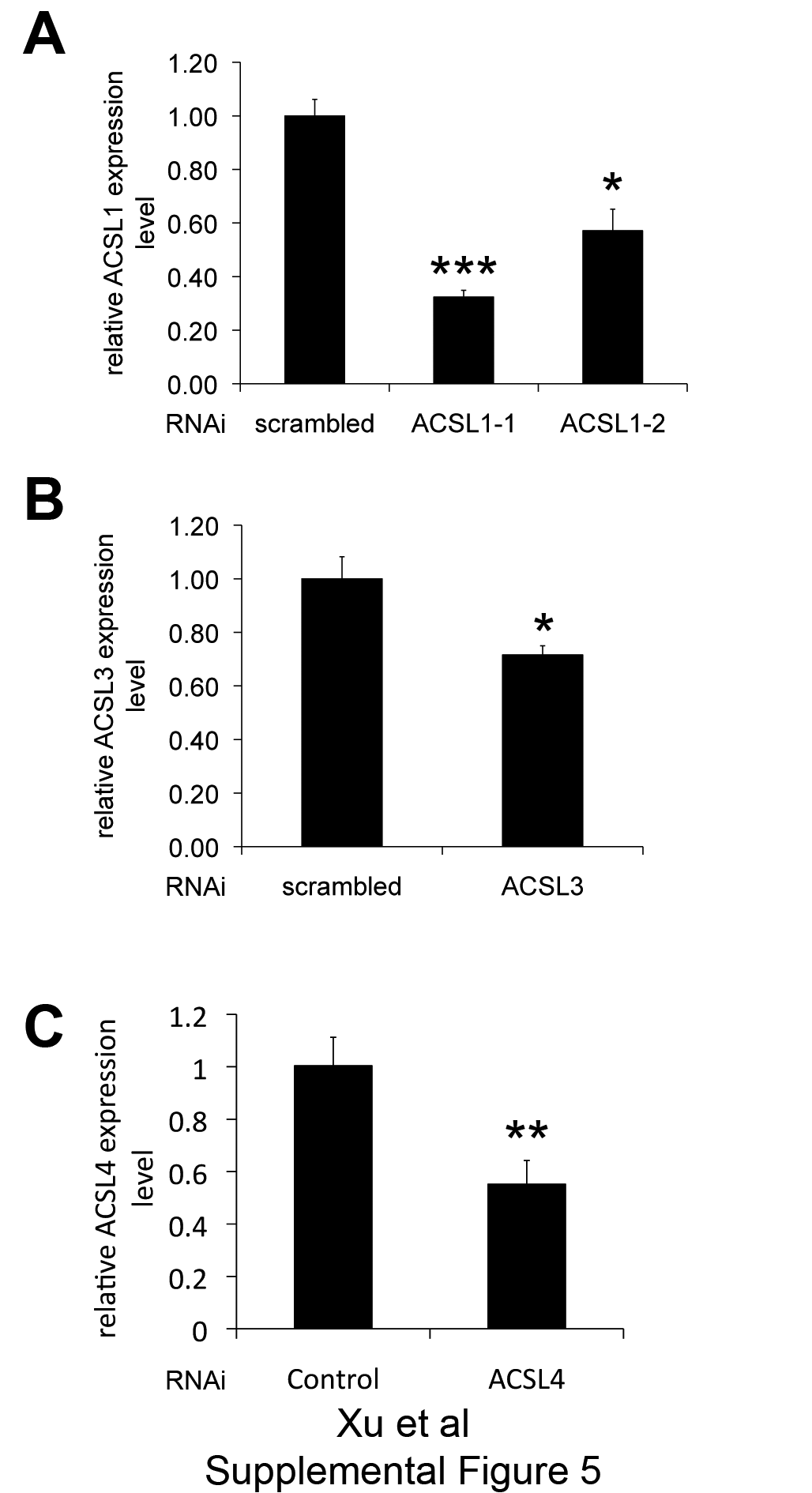

Supplement: Figure S5 — Knockdown efficiency of siRNAs for mouse ACSs. Knock-down efficiency in differentiated 3T3-L1s treated with siRNAs targeting ASCL1 (A) and ACSL3 (B) shortly prior to differentiation or with shRNA targeting ACSL4 (C). Targeted genes were analyzed by Q-PCR normalized to ß-actin. For all panels, assays done in triplicates. Error bars: Std. Dev., *ttest<0.05, **ttest<0.01, ***ttest<0.001. (TIF) [file pgen.1002478.s005.tif]
