# Supplementary material for: Insulin Signaling Regulates Fatty Acid Catabolism at the Level of CoA Activation
Source: PLoS Genet. 2012 Jan 19;8(1):e1002478. doi: 10.1371/journal.pgen.1002478 (PMC3261918; doi:10.1371/journal.pgen.1002478)
Supplement: Table S1 — Lipidomic profile of pudgy[BG] mutant and control animals. Lipid levels, in mg lipid per mg protein, in 3-day old control and pudgy[BG] mutant males. Average values and standard deviation for triplicate biological replicates are indicated, as well as the student t-test p-value indicating significance of the difference between controls and mutants. Values in parenthesis in the lipid names indicate the total number of carbons in the fatty acids chains, and the total level or desaturation. Ceramide (Cer), Cholesterol ester (ChoE), Diacylglycerol (DG), Lyso-Phosphatidylcholine (LysoPC), Monoacylglycerol (MG), Phosphatidic Acid (PA), Phosphatidylcholine (PC), Phosphatidylethanolamine (PE), Phosphatidylglycerol (PG), Phosphatidylserine (PS), Sphingomyelin (SM), Triacylglycerol (TG). (PDF) [file pgen.1002478.s006.pdf]

Supplemental Table 1: Lipidomic profile of *pudgy[BG]* mutant and control animals.

Lipid levels, in mg lipid per mg protein, in 3-day old control and *pudgy[BG]* mutant males. Average values and standard deviation for triplicate biological replicates are indicated, as well as the student t-test p-value indicating significance of the difference between controls and mutants. Values in parenthesis in the lipid names indicate the total number of carbons in the fatty acids chains, and the total level or desaturation. Ceramide (Cer), Cholesterol ester (ChoE), Diacylglycerol (DG), Lyso-Phosphatidylcholine (LysoPC), Monoacylglycerol (MG), Phosphatidic Acid (PA), Phosphatidylcholine (PC), Phosphatidylethanolamine (PE), Phosphatidylglycerol (PG), Phosphatidylserine (PS), Sphingomyelin (SM), Triacylglycerol (TG).

	Controls (Fed)		Mutants (Fed)		
**Name**	**average**	**stdev**	**average**	**stdev**	**ttest**
ChoE(19:0)	8.3E-04	1.4E-04	3.3E-03	9.2E-05	4.7E-05
TG(51:3)*	7.4E-03	8.2E-04	1.5E-02	6.2E-04	3.2E-04
TG(46:3)	9.6E-02	1.1E-02	2.1E-01	1.4E-02	4.8E-04
TG(53:3)	5.9E-03	5.4E-04	1.1E-02	2.9E-04	5.2E-04
TG(58:3)	1.5E-03	1.7E-04	3.1E-03	2.0E-04	5.8E-04
TG(14:0/16:0/18:1)	7.8E-01	1.4E-01	1.8E+00	1.1E-01	6.2E-04
TG(48:2)*	7.2E-01	7.9E-02	1.4E+00	1.0E-01	8.2E-04
TG(55:2)	3.1E-03	6.4E-04	9.5E-03	8.9E-04	8.8E-04
TG(49:1)*	4.8E-02	5.2E-03	1.1E-01	1.8E-03	1.0E-03
TG(40:2)	6.0E-03	2.7E-04	2.5E-02	1.3E-03	1.0E-03
TG(49:3)*	1.4E-02	2.0E-03	2.7E-02	1.7E-03	1.0E-03
TG(51:2)*	1.4E-02	1.2E-03	2.9E-02	2.2E-03	1.6E-03
LysoPC(20:5)	5.6E-03	4.0E-04	8.3E-03	4.5E-04	1.7E-03
TG(55:3)	3.0E-03	3.9E-04	6.1E-03	5.4E-04	1.8E-03
TG(53:4)	2.2E-03	3.8E-04	4.7E-03	2.1E-04	1.8E-03
TG(16:0/18:0/18:1)	6.2E-03	2.0E-04	1.6E-02	9.1E-04	1.9E-03
TG(16:0/18:1/18:1)	6.3E-02	1.3E-02	1.9E-01	3.5E-03	1.9E-03
TG(49:2)	4.1E-02	4.3E-03	7.9E-02	6.6E-03	2.1E-03
TG(48:4)*	5.3E-02	1.2E-02	1.2E-01	8.1E-03	2.4E-03
TG(14:0/18:1/18:1)+TG(16:0/16:1/18:1)	3.6E-01	1.1E-01	9.4E-01	7.7E-02	2.6E-03
TG(47:0)	3.3E-03	4.6E-04	7.3E-03	7.3E-04	2.6E-03
TG(47:3)	9.5E-04	1.5E-04	1.8E-03	1.8E-04	3.3E-03
TG(17:0/18:1/18:1)*	2.6E-03	2.8E-04	3.9E-03	2.5E-04	3.4E-03
TG(43:2)	8.7E-04	5.6E-05	2.5E-03	2.3E-04	4.5E-03
TG(58:2)	1.4E-03	2.9E-05	2.9E-03	1.9E-04	4.6E-03
TG(49:0)*	1.1E-03	6.9E-05	2.4E-03	2.1E-04	4.7E-03
DG(39:1e)	4.0E-04	1.6E-04	1.4E-03	2.3E-04	4.7E-03
PG(34:2)	3.7E-03	2.5E-04	5.1E-03	3.3E-04	5.3E-03
TG(16:0/18:2/18:1)	6.5E-02	2.2E-02	1.8E-01	1.0E-02	5.5E-03
TG(48:3)	3.2E-01	3.1E-02	7.2E-01	7.6E-02	5.5E-03
TG(50:3)*	2.3E-01	7.8E-02	6.6E-01	2.5E-02	6.4E-03
TG(50:1)	1.3E-01	1.9E-02	3.9E-01	5.1E-02	6.7E-03

TG(48:1)	2.5E-03	5.8E-04	4.6E-03	3.9E-04	9.2E-03
TG(53:1)	7.0E-03	1.7E-03	1.3E-02	1.4E-03	9.3E-03
TG(52:1)	2.7E-03	1.7E-04	4.8E-03	4.9E-04	1.0E-02
TG(41:1)	1.6E-03	3.5E-04	3.8E-03	6.0E-04	1.1E-02
TG(54:5)	3.8E-03	5.3E-04	6.1E-03	6.4E-04	1.1E-02
TG(46:1)*	1.2E+00	1.9E-01	2.2E+00	1.5E-02	1.1E-02
Cer(d18:0/24:0)	1.3E-03	1.0E-04	8.2E-04	1.4E-04	1.1E-02
TG(49:3)	3.1E-03	2.0E-04	4.8E-03	4.5E-04	1.2E-02
PC(36:3e)	1.3E-03	9.4E-05	8.2E-04	1.3E-04	1.2E-02
TG(53:3)	2.1E-03	3.9E-04	3.4E-03	2.0E-04	1.3E-02
TG(18:1/18:2/18:1)	7.3E-03	1.8E-03	1.4E-02	1.9E-03	1.4E-02
TG(51:4)	1.9E-03	3.3E-04	3.6E-03	5.2E-04	1.4E-02
TG(51:3)	4.8E-03	8.7E-04	7.6E-03	5.1E-04	1.5E-02
TG(53:2)	9.2E-03	9.2E-04	1.3E-02	3.3E-04	1.6E-02
PE(34:4e)	4.8E-03	9.3E-05	5.9E-03	3.0E-04	1.7E-02
TG(55:4)	1.4E-03	4.0E-04	2.7E-03	3.9E-04	1.7E-02
TG(40:1)*	1.0E-01	1.0E-02	1.6E-01	1.8E-02	1.8E-02
TG(39:1)	1.7E-03	4.6E-04	3.6E-03	6.7E-04	1.9E-02
PC(36:4)	7.3E-02	9.3E-03	1.0E-01	8.8E-03	2.0E-02
TG(46:0)	6.1E-03	1.1E-04	9.3E-03	8.5E-04	2.0E-02
PC(36:5)	1.0E-02	6.3E-04	1.2E-02	6.7E-04	2.0E-02
TG(54:1)*	1.6E-03	1.6E-04	2.6E-03	3.4E-04	2.3E-02
ChoE(26:3)	1.7E-03	3.7E-04	3.8E-03	7.6E-04	2.4E-02
TG(54:6)	2.6E-03	4.4E-04	3.9E-03	5.2E-04	2.6E-02
TG(51:2)	5.1E-03	9.5E-04	8.0E-03	3.0E-04	2.6E-02
TG(52:4)	3.8E-02	8.4E-03	1.0E-01	2.2E-02	2.7E-02
TG(14:0/16:0/18:0)	1.1E-02	2.7E-03	2.0E-02	7.5E-04	2.8E-02
ChoE(24:2)	3.6E-03	6.1E-04	6.8E-03	1.3E-03	3.0E-02
TG(51:3)	6.1E-03	5.4E-04	4.6E-03	5.1E-04	3.0E-02
PE(40:4)	5.8E-03	5.2E-04	4.3E-03	5.6E-04	3.0E-02
LysoPC(18:3)	3.3E-03	2.1E-04	3.8E-03	1.7E-04	3.5E-02
TG(46:2)*	9.2E-01	2.3E-01	1.5E+00	1.1E-01	3.5E-02
TG(47:1)	9.2E-02	8.2E-03	1.5E-01	2.3E-02	3.8E-02
PE(38:4)	1.5E-02	1.7E-03	1.0E-02	2.2E-03	4.0E-02
LysoPC(18:3)	1.2E-03	5.4E-05	2.1E-03	3.4E-04	4.1E-02
ChoE(26:2)	2.7E-02	2.5E-03	3.8E-02	4.7E-03	4.1E-02
PE(38:5e)	1.3E-02	1.5E-03	9.4E-03	1.6E-03	4.1E-02
TG(52:5)	1.2E-02	1.4E-03	3.0E-02	7.1E-03	4.6E-02
TG(50:2)	1.3E-03	2.4E-05	2.1E-03	3.3E-04	4.9E-02
TG(39:0)	2.6E-03	6.0E-04	3.9E-03	5.8E-04	5.1E-02
TG(56:3)	2.2E-03	3.3E-04	3.8E-03	7.7E-04	5.2E-02
TG(37:0)	3.0E-03	3.5E-04	4.4E-03	6.7E-04	5.5E-02
TG(51:1)*	9.9E-03	6.0E-04	2.3E-02	5.6E-03	5.6E-02
TG(44:0)*	2.3E-01	8.0E-03	3.4E-01	4.6E-02	5.6E-02
TG(38:0)*	2.2E-01	3.3E-02	2.8E-01	2.0E-02	5.7E-02
PE(36:2e)	8.4E-02	9.5E-03	6.2E-02	1.1E-02	6.1E-02
TG(56:2)*	4.2E-03	6.8E-04	6.8E-03	1.4E-03	6.5E-02
PC(34:1)	1.9E-02	1.9E-03	2.3E-02	1.1E-03	6.9E-02
TG(48:0)	8.5E-03	1.0E-03	1.0E-02	8.7E-04	7.1E-02
PC(36:3)	2.1E-02	1.9E-03	1.8E-02	1.9E-03	7.2E-02
TG(51:2)	8.7E-03	1.3E-03	6.3E-03	2.0E-04	7.3E-02
TG(50:0)	2.0E-03	6.7E-04	3.2E-03	3.4E-04	7.5E-02
PE(34:1e)	6.7E-03	4.6E-04	5.3E-03	8.0E-04	7.5E-02
TG(49:3)	4.1E-03	3.6E-04	3.5E-03	1.7E-04	7.7E-02
TG(42:1)*	3.8E-01	8.3E-02	5.4E-01	8.3E-02	7.7E-02
PG(34:1)	6.2E-04	1.7E-04	9.0E-04	5.5E-05	8.4E-02
PC(33:0)	8.4E-04	1.3E-04	1.1E-03	1.3E-04	8.5E-02
TG(51:4)	2.0E-03	4.4E-04	2.8E-03	5.3E-04	9.4E-02

LysoPC(14:0)	5.4E-04	8.8E-05	7.8E-04	1.5E-04	9.4E-02
LysoPC(18:2)	2.6E-02	4.7E-04	3.5E-02	5.3E-03	9.6E-02
TG(44:1)	8.8E-01	7.4E-02	1.2E+00	2.3E-01	1.0E-01
ChoE(26:2)	4.0E-03	2.3E-04	4.9E-03	6.3E-04	1.1E-01
PE(16:0p/18:1)	1.6E-02	2.9E-03	1.2E-02	7.8E-04	1.1E-01
LysoPC(18:4e)	2.3E-03	8.3E-04	1.0E-03	2.0E-04	1.2E-01
LysoPC(18:0)	7.4E-04	3.7E-05	6.8E-04	8.3E-06	1.2E-01
TG(43:1)	4.7E-03	9.2E-04	6.0E-03	5.8E-04	1.2E-01
PE(34:2)	6.2E-02	5.1E-03	9.8E-02	2.4E-02	1.2E-01
PE(34:2e)	6.1E-03	1.1E-03	4.5E-03	8.1E-04	1.2E-01
TG(18:1/18:1/18:1)	5.1E-03	8.9E-04	7.5E-03	1.7E-03	1.2E-01
TG(47:4)	1.1E-03	1.7E-04	1.7E-03	4.6E-04	1.3E-01
DG(33:5)	2.3E-03	1.4E-04	1.9E-03	3.1E-04	1.3E-01
Cer(d18:1/18:1)	8.5E-04	1.6E-04	6.3E-04	8.8E-05	1.3E-01
TG(16:0/18:1/20:1)+TG(18:0/18:1/18:1)	2.6E-03	5.9E-05	3.5E-03	6.2E-04	1.4E-01
TG(41:2)	1.3E-03	3.9E-04	2.9E-03	1.2E-03	1.4E-01
PE(36:5)	1.0E-02	1.3E-03	1.4E-02	3.2E-03	1.4E-01
DG(34:6e)	3.1E-03	5.5E-04	2.3E-03	4.0E-04	1.4E-01
MG(18:2)	8.6E-03	2.8E-04	1.2E-02	2.4E-03	1.5E-01
PE(30:2)	7.5E-03	1.5E-04	6.5E-03	7.6E-04	1.5E-01
MG(16:1)	8.4E-04	1.8E-04	6.0E-04	2.2E-05	1.6E-01
TG(48:5)	6.2E-03	1.8E-03	4.0E-03	2.3E-04	1.7E-01
PG(45:8)	4.6E-03	1.1E-03	3.3E-03	5.5E-04	1.7E-01
PG(36:2e)	3.8E-03	8.3E-04	2.9E-03	2.1E-04	1.8E-01
PC(36:4)	3.4E-03	4.1E-04	4.0E-03	4.7E-04	1.8E-01
PC(38:5)	1.9E-03	1.6E-04	1.6E-03	2.0E-04	1.8E-01
ChoE(25:2)	2.8E-01	9.8E-02	3.8E-01	5.5E-02	1.9E-01
ChoE(18:3)	9.1E-03	2.4E-03	6.5E-03	1.5E-03	1.9E-01
Cer(d18:1/16:0)	1.5E-03	1.3E-04	1.3E-03	2.4E-04	2.0E-01
SM(d18:1/13:0)	7.8E-03	1.6E-03	6.1E-03	5.5E-04	2.0E-01
TG(16:0/18:1/16:0)	1.9E-03	3.8E-04	2.3E-03	3.2E-04	2.1E-01
PE(36:3e)	9.7E-03	8.9E-04	8.8E-03	1.6E-04	2.2E-01
ChoE(20:4)	3.9E-03	1.1E-03	2.8E-03	4.6E-04	2.2E-01
PE(36:2e)	7.0E-03	1.5E-03	5.5E-03	1.4E-04	2.2E-01
PE(38:2)	1.8E-02	9.4E-04	2.0E-02	1.5E-03	2.2E-01
TG(54:0)	9.1E-03	5.8E-04	1.1E-02	1.5E-03	2.3E-01
PE(38:6)	9.1E-03	1.3E-03	7.4E-03	1.5E-03	2.3E-01
Cer(d18:1/18:0)	3.2E-03	4.9E-04	2.6E-03	4.8E-04	2.4E-01
PG(50:1e)	2.2E-03	3.3E-04	1.8E-03	4.0E-04	2.4E-01
TG(49:2)	4.4E-03	5.9E-04	5.2E-03	8.0E-04	2.4E-01
PE(34:1)	8.2E-02	5.5E-03	1.0E-01	2.0E-02	2.4E-01
PG(34:0)	8.1E-03	3.5E-04	7.0E-03	1.1E-03	2.4E-01
PE(38:2)	1.6E-02	2.6E-04	1.8E-02	2.4E-03	2.5E-01
PE(36:6e)	1.0E-02	1.5E-03	8.7E-03	1.2E-03	2.6E-01
PE(36:3)	3.7E-02	2.7E-03	3.9E-02	1.9E-03	2.6E-01
PE(38:6)	7.1E-02	9.2E-03	4.6E-02	2.9E-02	2.7E-01
PC(32:3e)	4.0E-03	8.0E-04	3.2E-03	6.0E-04	2.8E-01
LysoPC(20:4)	3.0E-03	3.7E-04	3.4E-03	4.7E-04	2.9E-01
PE(36:6e)	9.6E-02	1.3E-02	1.1E-01	5.7E-03	2.9E-01
PG(36:3)	4.6E-03	3.6E-04	4.9E-03	3.6E-04	2.9E-01
PE(32:0)	3.1E-03	3.9E-04	3.4E-03	4.1E-04	3.1E-01
PG(36:2)	2.5E-03	2.5E-04	2.2E-03	4.5E-04	3.1E-01
PG(43:7)	1.3E-03	1.2E-04	1.5E-03	3.2E-04	3.4E-01
PG(36:3e)	8.0E-03	1.8E-03	6.7E-03	9.4E-04	3.4E-01
PA(34:0e)	7.0E-03	1.0E-03	6.1E-03	9.4E-04	3.5E-01
SM(d18:1/19:3)	1.2E-03	9.3E-05	9.7E-04	2.6E-04	3.5E-01
Cer(d18:1/20:0)	1.0E-03	3.5E-04	7.9E-04	1.4E-04	3.5E-01
PE(36:4e)	5.0E-03	7.0E-04	5.8E-03	1.1E-03	3.6E-01

TG(49:2)	1.6E-02	1.8E-03	1.7E-02	1.2E-03	3.6E-01
PC(34:4e)	6.8E-02	1.1E-02	5.9E-02	1.2E-02	3.6E-01
PE(38:2)	1.0E-02	1.1E-03	9.1E-03	1.4E-03	3.7E-01
PE(38:5)	8.9E-03	2.5E-03	7.4E-03	8.5E-04	4.0E-01
PG(51:1e)	6.1E-03	1.1E-03	5.5E-03	4.0E-04	4.2E-01
SM(d18:1/17:1)	3.2E-03	7.8E-04	2.7E-03	5.5E-04	4.2E-01
PC(38:5e)	9.8E-04	1.8E-04	1.2E-03	2.8E-04	4.2E-01
PC(32:0)	3.6E-03	5.9E-04	3.9E-03	5.0E-04	4.3E-01
TG(41:0)	6.2E-03	7.8E-04	6.8E-03	9.7E-04	4.3E-01
PS(41:5)	2.6E-02	1.3E-03	2.5E-02	2.2E-03	4.3E-01
PC(34:3)	3.7E-02	5.2E-03	3.3E-02	6.1E-03	4.3E-01
PC(40:8)	3.7E-03	5.9E-04	3.3E-03	5.9E-04	4.4E-01
LysoPC(16:0)	8.4E-03	6.8E-04	8.9E-03	7.7E-04	4.5E-01
PE(38:6)	1.7E-02	6.5E-04	1.9E-02	2.5E-03	4.5E-01
TG(44:5)	2.0E-03	1.9E-04	2.3E-03	5.3E-04	4.6E-01
PS(32:0)	4.4E-03	2.9E-04	4.1E-03	4.8E-04	4.6E-01
PC(36:6e)	8.6E-04	8.9E-05	7.4E-04	2.3E-04	4.7E-01
PC(36:1)	2.1E-03	3.0E-04	2.4E-03	4.3E-04	4.9E-01
PE(34:4e)	6.7E-02	9.0E-03	7.3E-02	7.6E-03	5.0E-01
PE(36:1)	1.0E-02	6.1E-04	9.5E-03	1.7E-03	5.0E-01
PE(34:5)	5.7E-03	7.8E-04	5.2E-03	7.1E-04	5.0E-01
Cer(d18:1/18:0)	4.5E-03	5.2E-04	5.1E-03	1.1E-03	5.0E-01
PG(34:1e)	1.8E-03	2.4E-04	2.1E-03	5.5E-04	5.2E-01
PC(34:4)	2.3E-03	3.6E-04	2.6E-03	7.7E-04	5.2E-01
PC(34:2)	5.8E-02	7.5E-03	6.2E-02	6.9E-03	5.5E-01
PE(38:6e)	2.3E-02	2.1E-03	2.5E-02	3.6E-03	5.5E-01
PC(36:2)	9.7E-03	1.5E-03	9.1E-03	5.0E-04	5.6E-01
DG(28:0e)	8.7E-03	2.8E-03	7.5E-03	1.5E-03	5.8E-01
PA(34:0)	2.0E-02	5.1E-03	1.8E-02	4.0E-03	5.9E-01
TG(50:2)	7.1E-03	2.0E-03	6.4E-03	2.2E-04	5.9E-01
PE(36:3)	1.2E-01	7.1E-03	1.3E-01	3.3E-02	5.9E-01
Cer(d18:1/22:6)	3.9E-02	5.2E-03	3.7E-02	5.1E-03	6.4E-01
TG(40:0)*	7.5E-01	1.4E-01	8.0E-01	4.0E-02	6.5E-01
SM(d18:1/16:0)	2.3E-03	4.1E-04	2.1E-03	8.4E-04	6.5E-01
TG(42:0)*	8.1E-01	1.8E-01	7.6E-01	4.1E-02	6.7E-01
PE(34:3e)	1.2E-02	2.0E-03	1.3E-02	2.5E-03	6.8E-01
PE(34:4)	7.9E-03	6.2E-04	8.2E-03	9.7E-04	6.9E-01
DG(33:6e)	1.5E-03	5.1E-04	1.4E-03	2.1E-04	7.0E-01
TG(49:3)	2.1E-02	8.3E-03	1.9E-02	8.2E-04	7.2E-01
PE(36:3e)	2.0E-01	1.7E-02	2.0E-01	3.0E-02	7.2E-01
PC(38:7)	1.5E-02	1.5E-03	1.5E-02	2.9E-03	7.3E-01
PE(38:3e)	1.1E-02	6.5E-04	1.1E-02	2.3E-03	7.3E-01
PE(38:3e)	5.2E-03	7.5E-04	5.9E-03	3.1E-03	7.5E-01
LysoPC(16:1)	9.6E-03	1.3E-03	9.4E-03	2.7E-04	7.7E-01
PE(38:5)	8.3E-03	4.7E-04	8.2E-03	7.6E-04	8.0E-01
PC(32:3e)	9.5E-04	3.1E-04	9.0E-04	1.4E-04	8.0E-01
PG(43:8)	1.8E-02	3.6E-03	1.9E-02	3.3E-03	8.1E-01
PE(40:8e)	1.4E-02	9.5E-04	1.4E-02	1.5E-03	8.1E-01
PE(36:1)	8.8E-03	9.4E-04	9.1E-03	2.1E-03	8.4E-01
PA(34:0)	4.2E-02	4.8E-03	4.1E-02	1.0E-02	8.5E-01
PE(36:2)	5.2E-02	8.8E-03	5.1E-02	1.0E-02	8.6E-01
Cer(d18:1/18:1)	1.1E-03	1.6E-04	1.0E-03	3.4E-05	8.8E-01
PG(50:1e)	8.6E-03	1.9E-03	8.4E-03	1.4E-03	8.8E-01
PG(34:1)	5.2E-03	5.2E-04	5.3E-03	8.1E-04	8.8E-01
PC(38:2e)	1.5E-03	8.1E-05	1.6E-03	3.8E-04	8.8E-01
TG(53:4)	2.2E-03	3.7E-04	2.2E-03	1.8E-04	8.8E-01
PC(32:1)	2.7E-02	2.7E-03	2.7E-02	5.6E-03	9.0E-01
PC(34:0)	9.3E-02	2.8E-03	9.4E-02	8.8E-03	9.0E-01

TG(16:0/18:0/17:0)	2.2E-01	6.5E-03	2.2E-01	2.1E-02	9.0E-01
LysoPC(17:0)	1.7E-01	5.1E-03	1.7E-01	1.6E-02	9.0E-01
PE(34:0)	2.7E-01	8.1E-03	2.8E-01	2.6E-02	9.0E-01
Cer(d18:1/17:0)	1.7E-01	5.1E-03	1.7E-01	1.6E-02	9.0E-01
PG(45:6)	2.2E-02	5.7E-03	2.2E-02	7.0E-03	9.1E-01
PE(32:1)	5.7E-02	3.2E-03	5.6E-02	1.2E-02	9.3E-01
MG(16:1)	3.6E-03	4.1E-04	3.7E-03	4.0E-04	9.5E-01
PE(40:5)	5.4E-03	5.0E-04	5.4E-03	5.7E-04	9.5E-01
PC(38:6)	8.7E-03	5.6E-04	8.6E-03	4.1E-04	9.6E-01
TG(42:4)	4.1E-03	1.3E-04	4.1E-03	4.6E-04	9.7E-01
PC(36:3)	5.0E-02	5.1E-03	5.0E-02	2.4E-03	9.7E-01
Cer(d18:1/16:0)	2.6E-03	1.8E-04	2.6E-03	4.5E-04	9.8E-01
TG(47:2)	6.2E-03	3.9E-04	6.2E-03	2.3E-04	9.9E-01
ChoE(26:2)	2.8E-03	3.2E-04	2.8E-03	3.7E-04	1.0E+00
