# Supplementary material for: Insulin Signaling Regulates Fatty Acid Catabolism at the Level of CoA Activation
Source: PLoS Genet. 2012 Jan 19;8(1):e1002478. doi: 10.1371/journal.pgen.1002478 (PMC3261918; doi:10.1371/journal.pgen.1002478)
Supplement: Table S2 — TAG catabolism in pudgy[BG] mutant and control animals after 16 hours starvation. Lipid levels, in mg lipid per mg protein, in 3-day old control and pudgy[BG] mutant males starved for 0 or 16 hours. Average values and standard deviation for triplicate biological replicates are indicated. Values in parenthesis in the lipid names indicate the total number of carbons in the fatty acids chains, and the total level or desaturation. Ceramide (Cer), Cholesterol ester (ChoE), Diacylglycerol (DG), Lyso- Phosphatidylcholine (LysoPC), Monoacylglycerol (MG), Phosphatidic Acid (PA), Phosphatidylcholine (PC), Phosphatidylethanolamine (PE), Phosphatidylglycerol (PG), Phosphatidylserine (PS), Sphingomyelin (SM), Triacylglycerol (TG). (PDF) [file pgen.1002478.s007.pdf]

Supplemental Table 2: TAG catabolism in *pudgy[BG]* mutant and control animals after 16 hours starvation.

Lipid levels, in mg lipid per mg protein, in 3-day old control and *pudgy[BG]* mutant males starved for 0 or 16 hours. Average values and standard deviation for triplicate biological replicates are indicated. Values in parenthesis in the lipid names indicate the total number of carbons in the fatty acids chains, and the total level or desaturation. Ceramide (Cer), Cholesterol ester (ChoE), Diacylglycerol (DG), Lyso-Phosphatidylcholine (LysoPC), Monoacylglycerol (MG), Phosphatidic Acid (PA), Phosphatidylcholine (PC), Phosphatidylethanolamine (PE), Phosphatidylglycerol (PG), Phosphatidylserine (PS), Sphingomyelin (SM), Triacylglycerol (TG).

	**Averages**				**Stdev**			
	Controls		Mutants		Controls		Mutants	
**Name**	Fed	Starved	Fed	Starved	Fed	Starved	Fed	Starved
TG(46:1)*	1.2E+00	7.5E-01	2.2E+00	1.5E+00	1.9E-01	8.6E-02	1.5E-02	4.2E-01
TG(46:2)*	9.2E-01	5.8E-01	1.5E+00	1.1E+00	2.3E-01	9.1E-02	1.1E-01	2.4E-01
TG(44:1)	8.8E-01	5.6E-01	1.2E+00	1.0E+00	7.4E-02	1.1E-01	2.3E-01	2.7E-01
TG(42:0)*	8.1E-01	4.6E-01	7.6E-01	6.1E-01	1.8E-01	5.2E-02	4.1E-02	1.6E-01
TG(14:0/16:0/18:1)	7.8E-01	7.1E-01	1.8E+00	1.6E+00	1.4E-01	4.7E-02	1.1E-01	4.3E-01
TG(40:0)*	7.5E-01	4.3E-01	8.0E-01	5.7E-01	1.4E-01	2.2E-02	4.0E-02	1.7E-01
TG(48:2)*	7.2E-01	5.3E-01	1.4E+00	1.4E+00	7.9E-02	1.6E-01	1.0E-01	2.9E-01
TG(42:1)*	3.8E-01	2.6E-01	5.4E-01	4.2E-01	8.3E-02	4.2E-02	8.3E-02	1.5E-01
TG(14:0/18:1/18:1)+TG(16:0/16:1/18:1)	3.6E-01	3.1E-01	9.4E-01	7.9E-01	1.1E-01	1.0E-01	7.7E-02	2.3E-01
TG(48:3)	3.2E-01	2.6E-01	7.2E-01	5.9E-01	3.1E-02	6.4E-02	7.6E-02	1.3E-01
TG(44:0)*	2.3E-01	1.7E-01	3.4E-01	2.8E-01	8.0E-03	4.3E-02	4.6E-02	6.2E-02
TG(50:3)*	2.3E-01	1.9E-01	6.6E-01	5.4E-01	7.8E-02	6.9E-02	2.5E-02	1.3E-01
TG(38:0)*	2.2E-01	1.1E-01	2.8E-01	2.2E-01	3.3E-02	1.4E-02	2.0E-02	5.8E-02
TG(16:0/18:0/17:0)	2.2E-01	2.4E-01	2.2E-01	2.5E-01	6.5E-03	1.7E-02	2.1E-02	2.8E-03
TG(50:1)	1.3E-01	1.0E-01	3.9E-01	4.0E-01	1.9E-02	1.9E-02	5.1E-02	1.5E-01
TG(40:1)*	1.0E-01	5.0E-02	1.6E-01	1.1E-01	1.0E-02	2.3E-03	1.8E-02	2.9E-02
TG(46:3)	9.6E-02	6.3E-02	2.1E-01	2.1E-01	1.1E-02	1.0E-02	1.4E-02	7.7E-02
TG(47:1)	9.2E-02	6.0E-02	1.5E-01	1.5E-01	8.2E-03	5.8E-03	2.3E-02	5.0E-02
TG(16:0/18:2/18:1)	6.5E-02	5.7E-02	1.8E-01	1.7E-01	2.2E-02	1.7E-02	1.0E-02	3.6E-02
TG(16:0/18:1/18:1)	6.3E-02	4.8E-02	1.9E-01	1.8E-01	1.3E-02	1.6E-02	3.5E-03	6.9E-02
TG(48:4)*	5.3E-02	3.6E-02	1.2E-01	1.1E-01	1.2E-02	7.0E-03	8.1E-03	3.1E-02
TG(49:1)*	4.8E-02	3.2E-02	1.1E-01	8.4E-02	5.2E-03	4.6E-03	1.8E-03	3.3E-02
TG(49:2)	4.1E-02	2.7E-02	7.9E-02	6.5E-02	4.3E-03	2.6E-03	6.6E-03	9.5E-03
TG(52:4)	3.8E-02	3.8E-02	1.0E-01	1.1E-01	8.4E-03	1.4E-02	2.2E-02	5.6E-03
TG(49:3)	2.1E-02	1.9E-02	1.9E-02	1.7E-02	8.3E-03	1.5E-03	8.2E-04	6.2E-03
TG(49:2)	1.6E-02	1.6E-02	1.7E-02	1.7E-02	1.8E-03	1.4E-03	1.2E-03	4.1E-03
TG(49:3)*	1.4E-02	1.1E-02	2.7E-02	2.7E-02	2.0E-03	7.8E-04	1.7E-03	8.2E-03
TG(51:2)*	1.4E-02	1.2E-02	2.9E-02	2.7E-02	1.2E-03	1.9E-03	2.2E-03	8.3E-03

TG(52:5)	1.2E-02	1.0E-02	3.0E-02	2.7E-02	1.4E-03	3.7E-03	7.1E-03	7.4E-03
TG(14:0/16:0/18:0)	1.1E-02	1.1E-02	2.0E-02	1.9E-02	2.7E-03	5.5E-03	7.5E-04	6.3E-03
TG(51:1)*	9.9E-03	1.0E-02	2.3E-02	2.0E-02	6.0E-04	1.3E-03	5.6E-03	5.1E-03
TG(53:2)	9.2E-03	1.0E-02	1.3E-02	1.0E-02	9.2E-04	1.6E-03	3.3E-04	3.9E-03
TG(54:0)	9.1E-03	1.0E-02	1.1E-02	1.0E-02	5.8E-04	2.5E-03	1.5E-03	1.8E-03
TG(51:2)	8.7E-03	9.3E-03	6.3E-03	5.4E-03	1.3E-03	1.3E-03	2.0E-04	1.2E-03
TG(48:0)	8.5E-03	7.3E-03	1.0E-02	9.6E-03	1.0E-03	2.1E-03	8.7E-04	3.0E-03
TG(51:3)*	7.4E-03	7.7E-03	1.5E-02	1.4E-02	8.2E-04	9.2E-04	6.2E-04	3.5E-03
TG(18:1/18:2/18:1)	7.3E-03	7.8E-03	1.4E-02	1.5E-02	1.8E-03	2.0E-03	1.9E-03	5.3E-03
TG(50:2)	7.1E-03	6.5E-03	6.4E-03	5.6E-03	2.0E-03	3.6E-04	2.2E-04	2.2E-03
TG(53:1)	7.0E-03	6.4E-03	1.3E-02	1.4E-02	1.7E-03	1.2E-03	1.4E-03	3.5E-03
TG(41:0)	6.2E-03	6.5E-03	6.8E-03	5.9E-03	7.8E-04	8.4E-04	9.7E-04	1.6E-03
TG(16:0/18:0/18:1)	6.2E-03	5.2E-03	1.6E-02	1.3E-02	2.0E-04	4.0E-04	9.1E-04	5.2E-03
TG(47:2)	6.2E-03	5.2E-03	6.2E-03	5.8E-03	3.9E-04	1.5E-03	2.3E-04	1.1E-03
TG(48:5)	6.2E-03	5.2E-03	4.0E-03	5.1E-03	1.8E-03	6.5E-04	2.3E-04	8.4E-04
TG(46:0)	6.1E-03	5.5E-03	9.3E-03	8.6E-03	1.1E-04	8.2E-04	8.5E-04	1.6E-03
TG(51:3)	6.1E-03	8.4E-03	4.6E-03	5.3E-03	5.4E-04	2.5E-03	5.1E-04	1.1E-03
TG(40:2)	6.0E-03	4.2E-03	2.5E-02	2.1E-02	2.7E-04	1.2E-03	1.3E-03	3.2E-03
TG(53:3)	5.9E-03	6.3E-03	1.1E-02	7.6E-03	5.4E-04	1.2E-03	2.9E-04	1.9E-03
TG(18:1/18:1/18:1)	5.1E-03	6.1E-03	7.5E-03	8.6E-03	8.9E-04	2.8E-03	1.7E-03	2.7E-03
TG(51:2)	5.1E-03	4.4E-03	8.0E-03	6.9E-03	9.5E-04	2.9E-04	3.0E-04	2.6E-03
TG(51:3)	4.8E-03	4.6E-03	7.6E-03	6.2E-03	8.7E-04	1.2E-03	5.1E-04	1.9E-03
TG(43:1)	4.7E-03	3.3E-03	6.0E-03	5.5E-03	9.2E-04	4.1E-04	5.8E-04	1.2E-03
TG(49:2)	4.4E-03	5.3E-03	5.2E-03	5.5E-03	5.9E-04	5.7E-04	8.0E-04	1.9E-03
TG(56:2)*	4.2E-03	3.8E-03	6.8E-03	6.4E-03	6.8E-04	1.5E-03	1.4E-03	2.0E-03
TG(42:4)	4.1E-03	4.9E-03	4.1E-03	4.9E-03	1.3E-04	7.0E-04	4.6E-04	1.3E-03
TG(49:3)	4.1E-03	6.2E-03	3.5E-03	4.0E-03	3.6E-04	7.8E-04	1.7E-04	1.1E-03
TG(54:5)	3.8E-03	5.6E-03	6.1E-03	6.6E-03	5.3E-04	2.5E-03	6.4E-04	7.0E-04
TG(47:0)	3.3E-03	3.4E-03	7.3E-03	6.8E-03	4.6E-04	1.3E-03	7.3E-04	1.7E-03
TG(49:3)	3.1E-03	2.7E-03	4.8E-03	4.1E-03	2.0E-04	2.9E-04	4.5E-04	9.7E-04
TG(55:2)	3.1E-03	2.9E-03	9.5E-03	8.4E-03	6.4E-04	1.2E-03	8.9E-04	2.2E-03
TG(37:0)	3.0E-03	1.9E-03	4.4E-03	3.5E-03	3.5E-04	3.0E-04	6.7E-04	6.7E-04
TG(55:3)	3.0E-03	2.7E-03	6.1E-03	5.5E-03	3.9E-04	2.7E-04	5.4E-04	1.6E-03
TG(52:1)	2.7E-03	3.4E-03	4.8E-03	4.7E-03	1.7E-04	2.0E-03	4.9E-04	1.0E-03
TG(16:0/18:1/20:1)+TG(18:0/18:1/18:1)	2.6E-03	2.8E-03	3.5E-03	4.0E-03	5.9E-05	6.9E-04	6.2E-04	1.4E-03
TG(17:0/18:1/18:1)*	2.6E-03	2.6E-03	3.9E-03	3.9E-03	2.8E-04	3.9E-04	2.5E-04	1.9E-03
TG(39:0)	2.6E-03	1.6E-03	3.9E-03	3.4E-03	6.0E-04	9.7E-05	5.8E-04	8.1E-04
TG(54:6)	2.6E-03	2.7E-03	3.9E-03	3.1E-03	4.4E-04	8.3E-04	5.2E-04	5.6E-04
TG(48:1)	2.5E-03	2.4E-03	4.6E-03	4.2E-03	5.8E-04	5.9E-04	3.9E-04	1.2E-03
TG(56:3)	2.2E-03	1.7E-03	3.8E-03	3.5E-03	3.3E-04	2.9E-04	7.7E-04	1.2E-03
TG(53:4)	2.2E-03	2.5E-03	2.2E-03	2.8E-03	3.7E-04	2.6E-04	1.8E-04	8.6E-04
TG(53:4)	2.2E-03	2.3E-03	4.7E-03	4.1E-03	3.8E-04	7.1E-04	2.1E-04	1.1E-03
TG(53:3)	2.1E-03	2.4E-03	3.4E-03	3.5E-03	3.9E-04	1.5E-04	2.0E-04	1.1E-03
TG(44:5)	2.0E-03	2.5E-03	2.3E-03	2.5E-03	1.9E-04	7.5E-04	5.3E-04	4.0E-04
TG(50:0)	2.0E-03	2.4E-03	3.2E-03	2.7E-03	6.7E-04	1.3E-03	3.4E-04	9.5E-04
TG(51:4)	2.0E-03	2.7E-03	2.8E-03	1.8E-03	4.4E-04	2.2E-04	5.3E-04	3.6E-04
TG(51:4)	1.9E-03	1.8E-03	3.6E-03	4.2E-03	3.3E-04	1.8E-04	5.2E-04	1.6E-03
TG(16:0/18:1/16:0)	1.9E-03	4.4E-03	2.3E-03	4.5E-03	3.8E-04	6.1E-04	3.2E-04	1.4E-03
TG(39:1)	1.7E-03	1.1E-03	3.6E-03	3.5E-03	4.6E-04	2.9E-04	6.7E-04	4.7E-04
TG(54:1)*	1.6E-03	1.5E-03	2.6E-03	2.8E-03	1.6E-04	4.1E-04	3.4E-04	5.2E-04

TG(41:1)	1.6E-03	1.2E-03	3.8E-03	3.0E-03	3.5E-04	3.2E-04	6.0E-04	1.0E-03
TG(58:3)	1.5E-03	1.7E-03	3.1E-03	3.1E-03	1.7E-04	5.7E-04	2.0E-04	8.5E-04
TG(55:4)	1.4E-03	1.7E-03	2.7E-03	2.4E-03	4.0E-04	1.8E-04	3.9E-04	7.4E-04
TG(58:2)	1.4E-03	1.7E-03	2.9E-03	3.8E-03	2.9E-05	1.9E-04	1.9E-04	5.9E-04
TG(41:2)	1.3E-03	7.1E-04	2.9E-03	2.2E-03	3.9E-04	1.2E-04	1.2E-03	4.0E-04
TG(50:2)	1.3E-03	1.5E-03	2.1E-03	2.1E-03	2.4E-05	7.4E-04	3.3E-04	3.7E-04
TG(49:0)*	1.1E-03	1.5E-03	2.4E-03	1.9E-03	6.9E-05	1.3E-03	2.1E-04	9.5E-04
TG(47:4)	1.1E-03	2.3E-03	1.7E-03	2.9E-03	1.7E-04	3.7E-04	4.6E-04	6.7E-04
TG(47:3)	9.5E-04	9.3E-04	1.8E-03	2.2E-03	1.5E-04	1.2E-04	1.8E-04	7.3E-04
TG(43:2)	8.7E-04	6.1E-04	2.5E-03	1.9E-03	5.6E-05	1.1E-04	2.3E-04	3.1E-04
