# Supplementary material for: Insulin Signaling Regulates Fatty Acid Catabolism at the Level of CoA Activation
Source: PLoS Genet. 2012 Jan 19;8(1):e1002478. doi: 10.1371/journal.pgen.1002478 (PMC3261918; doi:10.1371/journal.pgen.1002478)
Supplement: Table S3 — Expression of mouse ACSs in Hepa1.6 and 3T3-L1 cells in response to serum removal (“−FBS”), or serum removal supplemented with insulin (“−FBS+insulin”) as in Figure 6 of the main text. (PDF) [file pgen.1002478.s008.pdf]

Supplemental Table 3A: Expression of mouse ACSs in Hepa1.6 and 3T3-L1 cells in response to serum removal ("-FBS"), or serum removal supplemented with insulin ("-FBS+insulin") as in Figure 6 of the main text.

		cell lines			
		Hepa1.6		3T3-L1	
genes	conditions	average	SD	average	SD
ACSM1	control	1.00	0.01	1.00	0.09
	-FBS	2.76	1.47	0.79	0.16
	-FBS+insulin	0.05	0.00	0.88	0.17
ACSM2	control	1.00	0.01	1.00	0.31
	-FBS	0.34	0.03	0.91	0.39
	-FBS+insulin	0.46	0.03	0.73	0.05
ACSM3	control	1.00	0.19	1.00	0.89
	-FBS	4.62	0.80	0.10	0.05
	-FBS+insulin	1.52	0.13	0.12	0.04
ACSM4	control	1.00	0.50	n.d	
	-FBS	0.89	0.53		
	-FBS+insulin	0.34	0.05		
ACSM5	control	n.d.		1.00	0.12
	-FBS			1.47	0.29
	-FBS+insulin			2.02	0.42
ACSF3	control	1.00	0.08	1.00	0.08
	-FBS	1.50	0.26	0.90	0.08
	-FBS+insulin	1.27	0.10	0.91	0.12
ACSL3	control	1.00	0.05	1.00	0.11
	-FBS	1.22	0.20	4.09	0.32
	-FBS+insulin	1.15	0.15	0.71	0.07
ACSL5	control	1.00	0.07	1.00	0.09
	-FBS	1.17	0.15	4.27	0.62
	-FBS+insulin	0.91	0.12	0.75	0.06
ACSL6	control	n.d.		1.00	0.06
	-FBS			0.10	0.01
	-FBS+insulin			0.15	0.03
ACSVL1	control	1.00	0.15	1.00	0.07
	-FBS	1.10	0.12	3.65	0.28
	-FBS+insulin	1.04	0.08	1.49	0.15
ACSVL2	control	n.d.		1.00	0.16
	-FBS			1.16	0.20
	-FBS+insulin			0.84	0.07
ACSVL3	control	1.00	0.05	1.00	0.13
	-FBS	1.27	0.22	1.85	0.17
	-FBS+insulin	0.82	0.09	1.26	0.23
ACSVL4	control	1.00	0.11	1.00	0.08
	-FBS	1.74	0.31	1.00	0.16
	-FBS+insulin	1.07	0.13	1.13	0.18
ACSVL5	control	1.00	0.02	1.00	0.06
	-FBS	13.10	3.06	0.26	0.03
	-FBS+insulin	1.59	0.17	0.26	0.05

Supplemental Table 3B: Expression of mouse ACSs in differentiated C2C12 cells in response to serum removal ("-FBS"), or serum removal supplemented with insulin ("-FBS+insulin") as in Figure 6 of the main text.

genes	conditions	average	SD
ACSM3	control	1.00	0.30
	-FBS	1.67	1.05
	-FBS+insulin	0.33	0.31
ACSF3	control	1.00	0.15
	-FBS	1.60	0.83
	-FBS+insulin	0.27	0.08
ACSL1	control	1.00	0.31
	-FBS	0.93	0.45
	-FBS+insulin	0.48	0.11
ACSL3	control	1.00	0.05
	-FBS	1.00	0.23
	-FBS+insulin	0.53	0.04
ACSL4	control	1.00	0.16
	-FBS	0.71	0.13
	-FBS+insulin	0.48	0.07
ACSL5	control	1.00	0.02
	-FBS	1.23	0.33
	-FBS+insulin	0.69	0.07
ACSL6	control	1.00	0.14
	-FBS	1.08	0.52
	-FBS+insulin	0.24	0.08
ACSVL1	control	1.00	0.64
	-FBS	2.26	1.04
	-FBS+insulin	0.87	0.98
ACSVL2	control	1.00	0.44
	-FBS	1.86	1.29
	-FBS+insulin	3.12	1.02
ACSVL4	control	1.00	0.26
	-FBS	1.44	0.69
	-FBS+insulin	0.20	0.08
ACSVL5	control	1.00	0.27
	-FBS	1.25	0.66
	-FBS+insulin	0.21	0.08
ACSVL6	control	1.00	0.20
	-FBS	1.32	0.61
	-FBS+insulin	0.20	0.08
