# Supplementary material for: Insulin Signaling Regulates Fatty Acid Catabolism at the Level of CoA Activation
Source: PLoS Genet. 2012 Jan 19;8(1):e1002478. doi: 10.1371/journal.pgen.1002478 (PMC3261918; doi:10.1371/journal.pgen.1002478)
Supplement: Text S1 — Supplemental Materials and Methods. (DOC) [file pgen.1002478.s009.doc]

**Supplemental Materials & Methods**

*Triglyceride quantification*

For triglyceride measurements, 6 flies or 4 larvae in triplicate were placed into Eppendorf tubes on ice, and then homogenized in 0.5 ml of 0.05% Tween-20 in d_2_H_2_O for 40 sec at 5000 rpm using a homogenizer with a plastic pestel. To measure protein levels, 100µl of lysate was cleared by centrifugation and 5 μl of the resulting supernatant was combined with 800 μl of ddH2O and 200 μL of Bio-Rad Protein Assay Reagent (catalog #500-0006), and incubated at room temperature for 3 min. OD595 was then measured and compared to a BSA standard curve. The remaining 400µl of fly homogenates were heat inactivated at 70 °C for 5 min, and then incubated with 10 μl of 10mg/mL lipoprotein-lipase from Chromobacterium viscosum (Sigma 62333) at 37 °C for 12h . Lysates were then spun at 5000 rpm on a tabletop centrifuge for 1 min, and 200 μl of the resulting supernatant was transferred to a fresh tube, avoiding debris. This supernatant was then spun at 14,000 rpm for 3 min in a tabletop centrifuge at 4°C. To measure triglycerides, 50 μl of the resulting supernatant was combined with 800 μl of free glycerol reagent (Sigma catalog #F6428) and incubated for 30 min at 37°C. OD540 was measured and compared with to a standard curve.

*Glycogen and trehalose quantification*

For glycogen measurement, 4 flies were homogenized in 400 μl 2% Na_2_SO4. 20 μl of the sample was then mixed with 46 μl 2% Na2SO4 and 934 μl chloroform/methanol (1:1), mixed, and spun at 14,000 rpm for 10 min. Supernatant was discarded and pellet was air dried in the hood for 10 min. 500 μl of anthrone reagent (0,2% (w/v) anthrone in 72% sulphuric acid) was then added to perform anthrone reaction (below).

For trehalose measurement, 4 flies were homogenized in 500 μl 70% ethanol. Samples were speed-vacuum dried to yield pellets that were then re-suspended with 200 μl 2% NaOH, heated at 100 C for 10 minutes, then cooled on ice. 100 μl of sample was then mixed with 750 μl anthrone reagent (0,2% (w/v) anthrone in 72% sulphuric acid) to perform an anthrone reaction.

Anthrone reaction: Mixtures were heated at 90 C for 20 minutes with mixing every 5 minutes, then cooled on ice for 10 minutes and then returned to room temperature for 20 minutes. The absorbance was read at 620 nm. The carbohydrate concentration was calculated using a standard curve.

*Starvation assay*

Growth controlled pdgy[BG] and w1118 control males were cultured on 0.8% agarose/PBS (30 flies per vial) at 25 C. Vials were changed and dead animals counted every ca. 4 hours.

*Longevity assay*

Growth controlled pdgy[BG] and w1118 control males were maintained on standard lab food. Vials were changed and dead animals were counted every 3-4 days.

*Lipidomic analysis: Total Lipids*

Freeze-dried Drosophila-samples (10.0-12.3 mg) were homogenized (20 Hz for 2 min) in Eppendorf-tubes with 200 µl of PBS-buffer by using zirconium oxide balls (one 5 mm and two 3 mm) in a Retsch mixer mill MM400 homogenizer (Retsch GmbH, Haan, Germany). For lipid profiling, a 5 µl aliquot was separated and spiked with an internal standard mixture consisting of 10 lipid classes at concentration levels of 0.2-0.6 µg/sample: GPCho(17:0/0:0), MG(17:0/0:0/0:0)[rac], DG(17:0/17:0/0:0)[rac], GPGro(17:0/17:0)[rac], Cer(d18:1/17:0), GPCho(17:0/17:0), GPA(17:0/17:0), GPEtn(17:0/17:0), DG(17:0/17:0/0:0)[rac] and TG(17:0/17:0/17:0). A 15 µl aliquot of PBS-buffer was added and the lipids were extracted with 100 µl of chloroform:methanol (2:1). The samples were vortexed for 2 min and after 30 min standing they were centrifuged at 10 000 rpm for 3 min. The lower lipid extracts were separated and another standard mixture containing 3 labelled lipid compounds (GPCho(16:1/0:0-D3), GPCho(16:1/16:1-D6), TG(16:0/16:0/16:0-13C3)) was added (0.1-0.2 µg/sample).

Protein content of the samples was determined from the homogenate (5 µl) which was diluted further for Micro BCA^TM^ Protein Assay Kit (Pierce, Rockford, IL, USA). Spectrophotometric determination was performed on a Multiskan EX instrument (Thermo Scientific Inc., Waltham, MA, USA). The lipid concentrations in the samples were normalised with the measured protein content.

The UPLC/MS system consisted of an Acquity Ultra Performance LC^TM^ (UPLC) combined with a Waters Q-Tof Premier mass spectrometer. A sample organizer is used for the automatic sampling.

The UPLC gradient started at 65% A / 35% B (AcCN / 2-Propanol, 1:1), and reached 80% B in 2 min, 100% B in 7 min and remained there for 7 min. The total run time including a 4 min re-equilibration step in initial conditions was 18 min. The temperature of the column (Acquity UPLC® BEH C_18_ 1.7 µm 2.1 × 100 mm; Waters Inc., Wexford, Ireland) was 50 °C, the flow rate 0.400 ml/min and the injected amount of lipid extract was 1 µl. The temperature of the sample organizer was 10 °C.

Lipids were detected by using electrospray ionization in positive ion mode. The data was collected in centroid mode at mass range of m/z 300-1200 with a scan duration of 0.2 s. Data was processed using the MZmine 2 software and lipids were identified using internal spectral libraries.

*Lipidomic Analysis: Free fatty acids*

10 mg tissue was weighted, 200 µl chloroform: methanol 0.1% BHT (2:1 v/v) was added together with 40 µl internal standard (heptadecantrienoat FFA C17:0 55 mg/l) and the sample was homogenized with zirconium oxide balls at 25 Hz 5 minutes with Mixer Mill 301 . 300 µl NaCl 0.9 % (0.15 M) was added to samples and the samples were vortexed for 2 min, incubated 30min at room temperature and the lower layer was separated by centrifuging at 10 000 rpm for 5 min. 800ul kloroform 0.1% BHT was then added to the samples and after vortex mixing (2 min) and incubation (30min at room temperature), the lower layer was separated by centrifuging at 10 000 rpm for 5 min.

The free fatty acids were cleaned by solid-phase extraction using Waters NH2 cartidges (1cc part WAT 023610). The SPE cartridges were conditioned with 2 x 1ml chloroform, 400 µl of the extract was mixed with 100 µl of chloroform and applied to the SPE cartridges. The cartridges were washed with 2 x 1ml chloroform, and the free fatty acids were eluted with 2 x 1 ml 2 % acetic acid in diethyl ether. The extractes were evaporated to dryness and silyated with 50ul MSTFA 1% trimethylchlorosilane (45 C, 1 h).

100 ul of the original extract was used for the determination of fatty acids. The extract was evaporated to dryness after addition of 10ul internal standard triheptadecanoic acid 582,5 mg/l. The evaporation residue was dissolved into petroleum ether (b.p. 40-60 °C; 700 µl). Fatty acids were transmethylated with sodium methoxide (NaOMe; 0.5 M; 250 µl) in dry methanol by boiling at 45 °C for 5 min. The mixture was acidified with 15% sodium hydrogen sulphate (NaHSO4; 500 µl) and the petroleum ether phase containing the fatty acid methyl esters (FAME) as well as free fatty acids (FFA) was collected. After centrifugation (10 000 rpm, 5 min) the petroleum ether layer was separated into a GC vial and evaporated, the residue was dissolved into hexane (100 µl) and 2 µl aliquot was used for GC analysis.

The samples (1 µl aliquots) were analysed on an Agilent 6890 gas chromatograph (GC) combined with Agilent 5973 mass selective detector (MSD). A splitless injection method was used and the injector and MSD interface temperatures were 260 °C and 280 °C, respectively. FFAs were analysed on an RTX-5 (5% phenyl methyl siloxane) capillary column (20m, ID 180 µm, film thickness 0.2 µm, Restek 40202) by using helium as carrier gas at constant flow mode (1.2 ml/min). FFAs were monitored at m/z 285 (14:0), 311 (16:1), 313 (16:0), 335 (18:3n-6), 337 (18:2n-6), 339 (18:1n-9), 335 (18:3n-3), 339 (18:1n-7), 341 (18:0), 361 (20:4n-6), 359 (20:5n-3), 363 (20:3n-6), 387 (22:5n-6), 385 (22:6n-3) and m/z 387 (22:5n-3). FAMEs were analysed with Agilent 5890 series II GC was equipped with a 25 m HP-FFAP Polyethylene Glycol TP: B006 column (25m. ID 200 µm, film thickness 0.3um) and helium was used as carrier gas. The temperatures of the GC injector (injection volume 1 μl) and detector (FID) were 260 and 300 °C, respectively, and the oven was programmed from 70 to 240 °C at 7 °C/min.

*Chromatin IPs*

Chromatin immunoprecipitation was performed as described before (Teleman et al., 2008). Quantitative PCR primer sequences are below.

*Cell culture*

Adipocytes were differentiated from murine 3T3-L1 as previously described (Bortell et al., 1992). Murine C2C12 skeletal myoblasts were cultured in DMEM with 10% FBS and their differentiation was induced using DMEM lacking FBS but supplemented with 2% horse serum. Hepa1.6 cells were cultured in DMEM supplemented with 10% serum. Cells were serum starved for 1 hour (3T3-L1) or for 4 hours (Hepa1.6 and C2C12), and then treated with or without insulin (5 μg/ml for 3T3-L1 and Hepa1.6, 100nM for C2C12) for 1 (3T3-L1 and Hepa1.6) or 2 hours (C2C12). Beta-actin served as normalization control.

*Generation of stable 3T3L1 cell line with ACSL4 Knock down.*

pLKO.1 Puro vectors carrying short hairpin RNA (shRNA) for ACSL4 (Sigma MISSION shRNAs) were cotransfected with the packaging plasmids PCMV-d78.74 and pMD2G into HEK293T/N17 cells. The media containing lentiviruses was collected 24h and 48h post transfection, centrifuged, filtered through a 0.45 µm filter and stored at -80^o^C. Undifferentiated 3T3L1 cells were infected with these combined viruses in DMEM medium. 24hr following infection, medium was changed to DMEM containing 3 µm/ml puromycin for selection of cells.

*Triglycerides analysis in differentiated 3T3-L1 cells treated with siRNA*

3T3-L1 cells were seeded at 90% confluence (Day 0) in 24-well plates with DMEM medium (1g/L glucose) supplemented with 10% FBS. On day 1 siRNAs were transfected using DharmaFECT.1 according to manufacturer’s instructions (Dharmacon). Final concentration for all siRNAs was 100nM. siRNA were from Ambion, and IDs and sequences are shown in the table below. On day 3, medium was replaced with differentiation medium 1 (DMEM-4.5g/L glucose with 10% FBS, 1µM Dexamethasone, 0.5 µM IBMX, 5 µg/ml insulin). On day 5, medium was replaced with medium 2 (DMEM-HG with 10% FBS, 5 µg/ml insulin) for 2 days and then maintained in DMEM-HG with 10% FBS for 3 more days. Cells were then lysed in 100 µl TN buffer (150mM NaCl, 50mM Tris pH7.5, 1% TritonX-100) on ice for 10 min. Cell lysates were collected and mixed by brief vortexing. Protein content was analyzed with Bradford assay and triglycerides were measured as described above.

siRNAs

| **gene** | **ID** | **sense** | **antisense** |
| --- | --- | --- | --- |
| ACSL1-1 | s65832 | CACUGAUGGUAUUCGAAGAtt | UCUUCGAAUACCAUCAGUGgt |
| ACSL1-2 | s65833 | GGAUGCUUCUCUUACUCAAtt | UUGAGUAAGAGAAGCAUCCtt |
| ACSL3 | s231894 | CACGUGAAAUUUUAAAUGAtt | UCAUUUAAAAUUUCACGUGtt |

*Luciferase assay*

As previously described (Teleman et al., 2008), test firefly luciferase constructs were co-transfected with a normalization control construct (renilla luciferase under control of a basal Adh promoter) together with a copper-inducible FOXO expression construct (pMT-FOXO) into S2 cells grown in Schneider’s medium supplemented with 10% FCS, using Effectene reagent (Qiagen). Expression of FOXO was either induced or not via addition of cupric sulfate, and luciferase activity measured as described (Teleman et al., 2008). All the experiments were performed in triplicate. Firefly luciferase activity was normalized by the renilla value in each well.

*Food intake assay*

Six, male, growth-controlled, 3^rd^ instar larvae (96h AEL) or adults were kept on food supplemented with 0.8% Blue9 (erioglaucine disodium salt) for 20 minutes, or 2 hours respectively. Animals were then homogenized in PBS, lysates were spun at 14,000 rpm on a tabletop centrifuge for 20 min, and 200 μl of the resulting supernatant was transferred to a 96-well plate, avoiding debris. Absorbance at 625 nm was recorded by Tecan plate-reader. For blank measurements, equivalent lysates were prepared from animals fed on food without dye.

*Nile red staining*

Growth controlled early L3 larvae were starved on 0.8% agarose/PBS for 0 or 24 hours, Fat bodies were stained with Nile red as described (Palanker et al., 2009).

*Immunofluorescence staining*

S2 cells were transfected with pMT-pudgy-HA and Tubulin:mito-GFP on glass coverslips. Twenty-four hours after induction with copper, the cover slips were rinsed with PBS once and fixed for 15 min with 4% paraformaldehyde in PBS warmed to room temperature. The cover slips were rinsed twice with PBS and cells were permeabilized with 0.2% Triton X-100 in PBS for 5 min. After rinsing twice with PBS, the cover slips were blocked with 5% BSA in PBS for 30 min, then incubated with primary antibody in 5% BSA in PBS with 0.2% triton X-100 for 2 hr at room temperature, then rinsed four times with PBS, and then incubated with secondary antibodies produced in donkey (diluted 1:1000 in 5% BSA with 02.% triton X-100) for 40 min at room temperature in the dark. After washing four times with PBS, cover slips were mounted onto slides and imaged with a Leica SP5 confocol microscope.

*Colocalization analysis on immunofluorescence micrographs*

Co-localization analysis was performed on 8 independent images of S2 cells stained for pudgy-HA and mito-GFP using the using the JACoP plugin (Bolte et al., 2006) for ImageJ.

*Quantitative RT–PCR*

Total RNA was obtained from larval tissues or adult flies by homogenization in TRIzol reagent (Invitrogen catalog #15596-018). Two micrograms of total RNA from each sample was used to generate first-strand cDNA using the SuperScript III kit (Invitrogen 11752-250) using the poly(dT) method. Quantitative RT–PCR was then done on the Applied Biosystems ABI StepOnePlus Real-time RT–PCR system using the SYBR Green PCR master mix (ABI catalog #4309155). All assays were done in triplicate and normalized to rp49 levels, and errors were propagated in all calculations.

*Oligos*

Oligos for Genomic Quantitative-PCR (for Chromatin IPs)

| **gene** | **5' oligo** | **3' oligo** |
| --- | --- | --- |
| 4E-BP | CGGCAATAACAACAAGAACC | GGTCGGCTGATGTTTTTTG |
| mir278 | CGGGTCGGTGGGACTTTC | GACGGCAGGTGCAAGACAG |
| sty | AATCGGCGAGGATGAGTG | TGGCACAGCGAACAAAGT |
| P1 | TCTCCATCGCTGTGTAAACA | CCCGAGCCAATTGTTGAATA |
| P2 | CCTTTTGGCCGTAATAGTG | TGACGGGTTGTGCTGATAA |

Oligos for Quantitative RT-PCR on selected genes.

Additional oligo sequences available upon request.

| **gene** | **5' oligo** | **3' oligo** |
| --- | --- | --- |
| CG9009 | CGGGACTGACGGTGACCA | CGGATACCGTTCCCACCA |
| 4EBP | CACCACTCCTGGAGGCACCAA | GAAGGGAGTACGCGGAGTTC |
| rp49 | GCTAAGCTGTCGCACAAA | TCCGGTGGGCAGCATGTG |
| mAcsm1 | GATTTGTGGCCTGCAGCAA | GCATGCAGCCCACTGTCA |
| mAcsm2 | CCCAGGAAGGTGGAGTTCGT | CGAAGCTTTGCTCGCTCAAT |
| mAcsm3 | GCACACTATTTGCCCCGTTT | TAGGCAGTTGGTGCAGAACAGA |
| mAcsm4 | TTCTGGACCAGTGGTCCCTAA | CCTCATCTCCTTTCCCATTCAC |
| mAcsm5 | TGTCCCTCCCTCCAAACCA | AAGCCGCTCTCAGGAGTTCTC |
| mAcsf3 | CTGAGCACCCACCGTAACCT | TTTTTAGTCCACGCCCATGAG |
| ACSL1 | GTATGCCACCAGGCCTAAGG | CAGTGGTACCCGCTATTTCCA |
| ACSL3 | TCTTGCAAACAAAGCTGAAGGA | AGGCTTCCCATCAACAGTAATGA |
| ACSL4 | ACCATTGCCATTTTCTGTGAGA | GTGGCATATAAAGTCACAAGTGGAA |
| ACSL5 | TCCTCAAATTTCTGGAGCCTATCT | TGAGCCAAGGGAAGGTAGGAT |
| ACSL6 | CTCCTGAAGCAGTCGGAAGAA | TGCAACCCCCAATCACAGA |
| ACSVL1 | TGTGCAACACACCGCAGAA | GCCATTTCCCAGGGCTTTT |
| ACSVL2 | GGTGGTGGGCGGAGATTT | GAATCCTTCATTCCCCAAACTCT |
| ACSVL3 | CCCCTTCCTGGGCTATGC | AAAACGTCCCCAGACCAGAA |
| ACSVL4 | CCACTCAGCAGGAAACATCGT | CTTCCGGATCACCACAGTCA |
| ACSVL5 | CGAGGCGAGACCTCTTTGG | CGGTGTCGTCGCAGCTCTA |
| ACSVL6 | GGGCCAGTGTGCTGATTGT | TTGTATGACCCGCTCATGTGA |
| ACTB | GGCTGTATTCCCCTCCATCG | CCAGTTGGTAACAATGCCATGT |

Cloning oligos

| **cloning** | **5' oligo** | **3' oligo** |
| --- | --- | --- |
| mito-GFP | GTTGGTACCATGTCCGTCCTGACGCCGCT | GGGTCTAGATTACTTGTACAGCTCGTCCA |
| pUAST-CG9009 | GGGCTCGAGATGAACCCAGCCGCTCAA | GGGTCTAGATTAATCGGAGAACTTCTC |
| CG9009-HA | gggGGTACCATGAACCCAGCCGCTCAA | GGGGTCGACTTACGGCGTAGTCGGGCACGTCGTAGGGGTAGGATCCATCGGAGAACTTCTCCTT |
| CG9009>>Luc | GAGGGTACCAATTTGGAACGCAGAGAA | GGGGGTACCAAGTACAAAGTTCGCCTT |

*References*

Bolte, S. and Cordelieres, F.P. (2006) A guided tour into subcellular colocalization analysis in light microscopy. J Microsc 224: 213-232.

Bortell, R., van Wijnen, A.J., Ramsey-Ewing, A.L., Stein, G.S., and Stein, J.L. (1992). Differential regulation of H4 histone gene expression in 3T3-L1 pre-adipocytes during arrest of proliferation following contact inhibition or differentiation and its modulation by TGF beta 1. Journal of cellular biochemistry 50, 62-72.

Palanker, L., Tennessen, J.M., Lam, G., Thummel, C.S. (2009) Drosophila HNF4 regulates lipid mobilization and beta-oxidation. Cell metabolism 9, 228-239.

Teleman, A.A., Hietakangas, V., Sayadian, A.C., and Cohen, S.M. (2008). Nutritional control of protein biosynthetic capacity by insulin via Myc in Drosophila. Cell metabolism 7, 21-32.
